# Deciphering molecular pathways in urological cancers: A gateway to precision therapeutics

**DOI:** 10.1016/j.jare.2025.06.009

**Published:** 2025-06-12

**Authors:** Kiavash Hushmandi, Najma Farahani, Behzad Einollahi, Shokooh Salimimoghadam, Mina Alimohammadi, Liping Liang, Le Liu, Gautam Sethi

**Affiliations:** a Nephrology and Urology Research Center, Clinical Sciences Institute, Baqiyatallah University of Medical Sciences, Tehran, Iran; b Farhikhtegan Medical Convergence sciences Research Center, Farhikhtegan Hospital,TMs.C., Islamic Azad University, Tehran, Iran; c Department of Biochemistry and Molecular Biology, Faculty of Veterinary Medicine, Shahid Chamran University of Ahvaz, Ahvaz, Iran; d Department of Immunology, School of Medicine, Shahid Beheshti University of Medical Sciences, Tehran, Iran; e Guangzhou Key Laboratory of Digestive Diseases, Department of Gastroenterology and Hepatology, Guangzhou Digestive Disease Center, Guangzhou First People’s Hospital, School of Medicine, South China University of Technology, Guangzhou 510180, China; f Department of Gastroenterology, Zhujiang Hospital, Southern Medical University, Guangzhou 510280, China; g Department of Pharmacology and NUS Centre for Cancer Research (N2CR), Yong Loo Lin School of Medicine, National University of Singapore 117600 Singapore, Singapore

**Keywords:** Urologic neoplasms, Targeted therapy, Therapeutic resistance, Precision oncology, Biomarker-driven treatment

## Abstract

**Background:**

Urological cancers, including prostate, kidney, bladder, testicular, and penile cancers, pose a significant health challenge, particularly in their metastatic stages. Surgical interventions remain fundamental, but recent advancements in medical therapies like chemotherapy, immunotherapy, and targeted therapies have shown promise in improving patient outcomes.

**Aim of review:**

This review aims to explore the current landscape of targeted therapies in urological cancers, focusing on the role of key signaling pathways such as phosphatidylinositol 3-kinase/protein kinase B (PI3K/Akt), mechanistic (mammalian) target of rapamycin (mTOR), growth factor-related mechanisms, c-Mesenchymal-epithelial transition factor (c-Met)/hepatocyte growth factor (HGF), programmed cell death protein 1 and its ligand programmed death-ligand 1 (PD-1/PD-L1), and steroid hormone receptor pathways in tumor progression and therapeutic resistance.

Key scientific concepts of review

Dysregulation of pathways like PI3K/Akt and mTOR contributes to tumorigenesis, metastasis, and resistance to treatment, underscoring their relevance as therapeutic targets. Tyrosine kinase inhibitors and immune checkpoint inhibitors have demonstrated efficacy but face challenges such as intrinsic resistance and treatment-related toxicities. Integrating insights from signaling pathway research with clinical practice holds potential for developing more effective treatment paradigms, enhancing the efficacy of targeted therapies, and improving survival rates for patients with urological cancers.

## Highlights

• Urological cancers include prostate, kidney, bladder, testicular, and penile malignancies.
• Surgical intervention is often the primary treatment for urological cancers.
• Innovative therapies such as immunotherapy and targeted treatments enhance patient outcomes.
• Key signaling pathways drive tumor progression and resistance in urological cancers.
• Understanding molecular networks is vital for improving targeted therapy effectiveness.

## Graphical abstract

**Figure:**
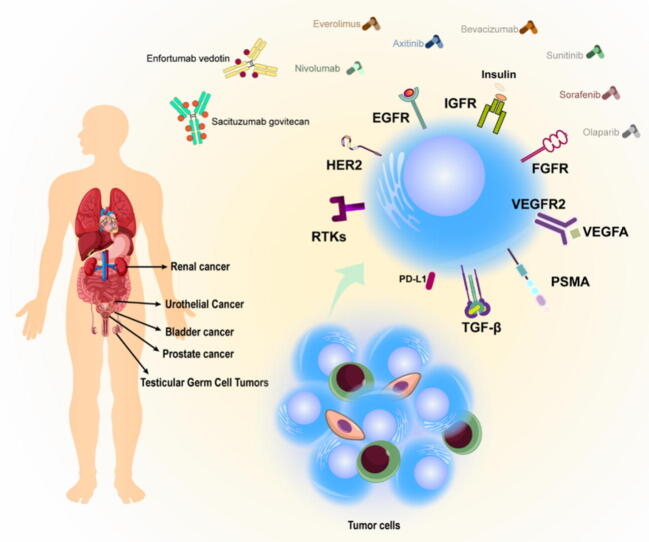

## Introduction

Urological malignancies are typically categorized to include cancers of the prostate, kidney, bladder, testicles, and penis. Prostate, testicular, and penile cancers are exclusive to males, whereas kidney and bladder cancers can develop in both genders, though males are at a higher risk. In recent years, numerous innovative treatment approaches have been introduced to manage these cancers and enhance patient survival rates. However, effectively treating these cancers remains difficult, particularly when they progress to a metastatic stage, with prostate cancer presenting significant challenges [1]. Urological cancers, including bladder and prostate cancer, continue to represent a significant global health burden due to their high incidence and mortality rates. According to the Global Cancer Observatory (GCO) by the World Health Organization (WHO) and the International Agency for Research on Cancer (IARC), bladder cancer accounted for over 600,000 new cases and approximately 220,000 deaths globally in 2022, while prostate cancer had more than 1.4 million new cases. Recent clinical trials have demonstrated promising advances in treatment. For instance, the TAR-200 drug delivery system for bladder cancer has shown encouraging clinical responses, with objective response rates reaching approximately 83 %, thereby providing valuable data on the safety and efficacy of novel intervention [2]. Surgical intervention is frequently the mainstay treatment for urological cancers, with the specific surgical method tailored to the type and stage of the cancer. Beyond surgery, a range of medical treatments are utilized, including chemotherapy, which is often combined with surgical procedures or used as the primary treatment for advanced stages of cancer. Chemotherapy can be delivered systemically or directly into the bladder through intravesical administration. Another therapeutic option is immunotherapy, which leverages the body’s immune system to target and destroy cancer cells [3]. For instance, Bacillus Calmette-Guérin (BCG) is primarily employed in the treatment of bladder cancer. Additionally, targeted therapies are available that concentrate on specific molecular targets within cancer cells, thereby reducing harm to healthy tissues. Signaling pathways are integral to the initiation and progression of urological tumors. Several pathways have been identified that facilitate tumor growth, spread, and resistance to treatments. Notable examples include the hepatocyte growth factor (HGF)/c-Mesenchymal-epithelial transition factor (c-Met) pathway, the programmed cell death protein-1/programmed death-ligand 1 (PD-1/PD-L1), steroid hormone receptor, and calcium signaling pathways, among others, which play significant roles in the advancement of urological cancers [4]. Recent progress in targeted therapies, such as tyrosine kinase inhibitors and immune checkpoint inhibitors, shows potential in enhancing patient outcomes. Nonetheless, issues like inherent resistance and treatment-related side effects highlight the need for a more comprehensive understanding of these molecular networks [5]. This review seeks to shed light on the complex signaling pathways involved in urological cancers, aiming to inform novel therapeutic strategies that could improve the effectiveness of targeted treatments and ultimately increase survival rates for affected individuals.

## Understanding signaling pathways and targeting them for cancer therapy

### Overview of key signaling pathways

Extensive research has documented genetic alterations associated with cancer, beginning with the discovery of key oncogenes such as MYC (v-myc avian myelocytomatosis viral oncogene homolog), RAS (rat sarcoma virus oncogene), BRAF (v-raf murine sarcoma viral oncogene homolog B1), and KIT (v-kit Hardy-Zuckerman 4 feline sarcoma viral oncogene homolog). These discoveries were complemented by the identification of critical tumor suppressor genes, including TP53 (tumor protein p53), BRCA1 (breast cancer type 1 susceptibility gene), and PTEN (phosphatase and tensin homolog), all of which play essential roles in maintaining genomic stability and regulating cell proliferation. Current understanding emphasizes how molecular networks and signaling pathways regulate essential cell survival and growth processes, making them central to both cancer development and therapeutic approaches. This discussion examines specific signaling pathways and cancer types where targeted treatments have enhanced patient outcomes. Notably, two frequently dysregulated pathways in cancer are the phosphatidylinositol 3-kinase/protein kinase B/mechanistic target of rapamycin (PI3K/AKT/mTOR) pathway and the RAS/mitogen-activated protein kinase (RAS/MAPK) pathway [6]. These systems work together to transmit signals from receptor tyrosine kinases to cellular regulators and effector proteins. Beyond their direct signal transmission, these pathways interact through various feedback mechanisms. For instance, inhibition of mechanistic target of rapamycin complex 1 (mTORC1) using rapamycin analogs (rapalogs) can lead to compensatory reactivation of the MAPK pathway, potentially contributing to therapeutic resistance. Studies have demonstrated that co-targeting both the mTOR and MAPK signaling pathways yields more effective therapeutic outcomes. Cancer is often characterized by dysregulation of pathways involved in cell cycle regulation and the maintenance of genomic stability. Comprehensive analyses across multiple cancer types have shown that genomic instability frequently results in early chromosomal aberrations, which play a pivotal role in the pathogenesis of cancers such as glioblastoma, melanoma, and breast adenocarcinoma.

Research shows that targeting both pathways simultaneously produces better therapeutic results. Cancer frequently involves alterations in pathways controlling cell cycle progression and DNA stability maintenance. Analysis across multiple cancer types reveals that genomic instability leads to early chromosomal changes in various cancers, including glioblastoma, melanoma, and breast adenocarcinoma [7]. This understanding has led to clinical trials testing cyclin-dependent kinases 4 and 6 (CDK4/6) as well as poly (ADP-ribose) polymerase (PARP) inhibitors, particularly for cancers with DNA repair deficiencies. Hormone-dependent cancers, such as breast, ovarian, and prostate cancers, rely heavily on hormone-stimulated growth. Understanding hormone receptor mechanisms has enabled the development of various inhibitors now standard in cancer treatment. Beyond these examples, therapeutic success has been achieved through targeting other mechanisms, including apoptotic pathways. New research indicates that β-adrenergic signaling, both in the broader tumor environment and within tumor tissues, influences cancer progression and can be targeted with β-blockers [8]. While decades of research have identified crucial cancer-related alterations and improved treatment options, leading to better survival rates, even advanced targeted therapies often fail to achieve complete cancer remission.

### Cancer targeted therapy directed at signaling pathways

#### PI3K/Akt signaling

**Fig. 1:**
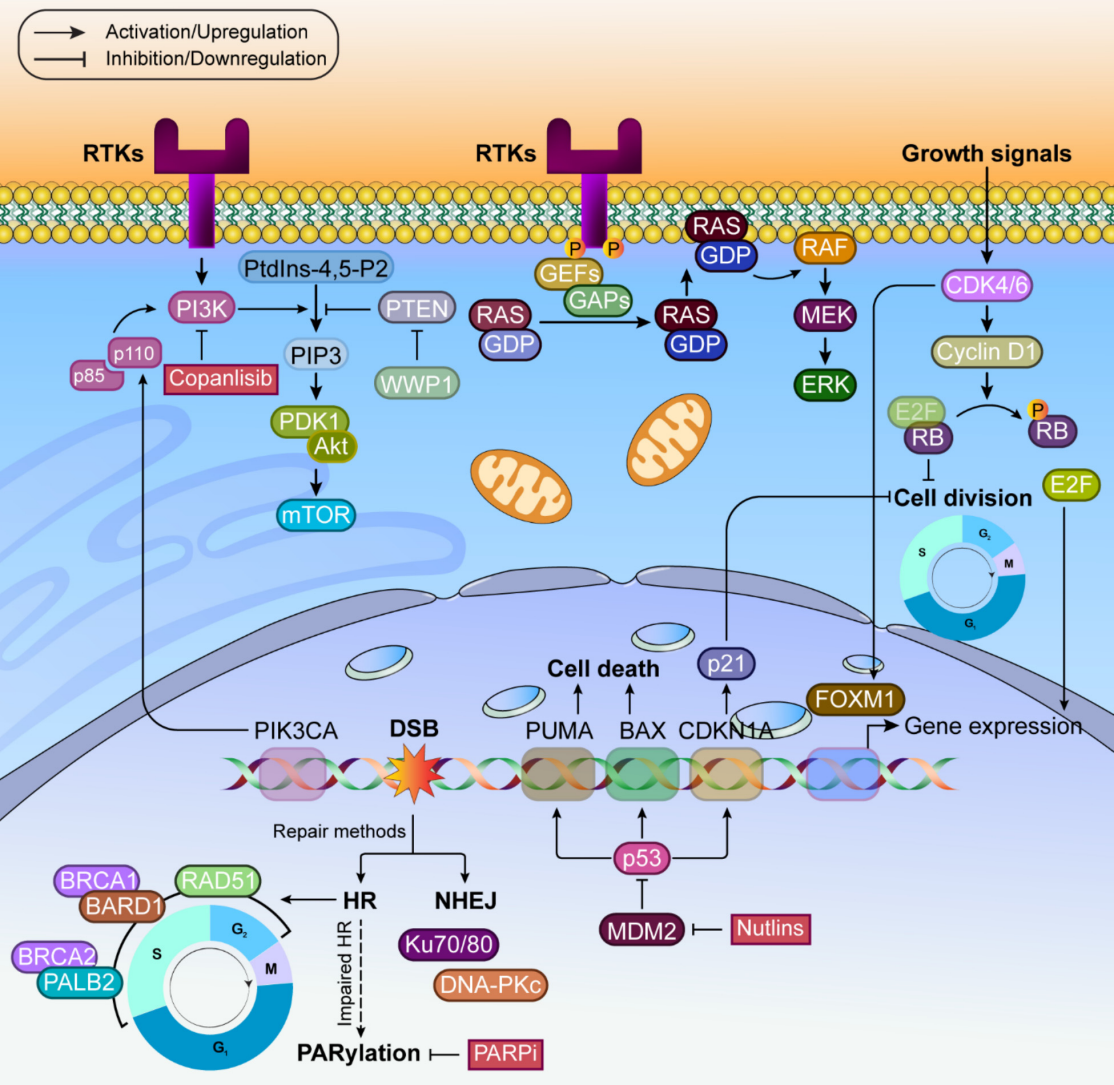
Major cancer targeted therapies directed signaling pathways.

Several cancer targeted therapies based on signaling pathways have been realized to date (Please refer to Fig. 1). The PI3K signaling pathway is crucial for regulating cellular growth, survival, and proliferation in response to various receptor tyrosine kinases (RTKs). Among several PI3K classes, class IA is most commonly altered in cancer. This class consists of p110 (catalytic) and p85 (regulatory) subunits. Upon activation by RTKs or adaptor proteins, PI3K catalyzes the conversion of phosphatidylinositol 4,5-bisphosphate (PtdIns-4,5-P2) into phosphatidylinositol 3,4,5-trisphosphate (PIP3). PIP3 subsequently activates 3-phosphoinositide-dependent protein kinase-1 (PDK1) and mechanistic target of rapamycin complex 2 (mTORC2). These kinases phosphorylate protein kinase B (AKT), a central signaling protein that regulates multiple processes related to cell survival, proliferation, and metabolism. This pathway is tightly controlled by several tumor suppressors, including PTEN, promyelocytic leukemia protein (PML), and tuberous sclerosis complex (TSC). In cancer, mutations in PIK3CA (which encodes the p110α catalytic subunit of PI3K) and PTEN are particularly significant [9]. PTEN alterations appear in many cancers and can be either inherited or acquired. Research using mouse models has shown that certain PTEN mutations can interfere with normal PTEN function more severely than simple PTEN reduction. Early research with PI3K inhibitors showed promise, but many broad-spectrum inhibitors failed in clinical trials due to toxicity. Copanlisib remains an exception, gaining the U.S. Food and Drug Administration (FDA) approval for certain lymphomas. More recent approaches focus on targeting specific PI3K forms. Notable successes include idelalisib for leukemia and alpelisib for certain breast cancers. Newer drugs like taselisib and GDC-0077 show promise in ongoing trials. Treatment resistance often develops through various mechanisms, including alternate signaling pathways and PI3K isoform activation. Combined targeting of different PI3K forms has shown better results in some cases. The co-occurrence of PIK3CA mutations with other genetic changes can affect treatment outcomes, as seen in studies of breast cancer patients [10]. Recent research has explored reactivating remaining functional PTEN in cancers with partial PTEN loss. For instance, targeting the WW domain-containing E3 ubiquitin protein ligase 1 (WWP1) enzyme, which inhibits PTEN, has shown promise in certain cancer models. AKT, a key downstream component, exists in three forms and promotes cell survival. Its overactivation appears in many cancers and often contributes to treatment resistance. Inhibitors like ipatasertib show particular effectiveness in PTEN-deficient cancers. Current clinical trials are examining various combination approaches using AKT inhibitors. This simplified overview of the complex PI3K pathway demonstrates how understanding molecular mechanisms can lead to targeted cancer treatments, though challenges remain in preventing resistance and optimizing therapeutic approaches [11].

#### Ras/MAPK signaling

When RTKs become active, they trigger the activation of Ras proteins by promoting their transition from an inactive guanosine diphosphate (GDP)-bound state to an active guanosine triphosphate (GTP)-bound state. This activation is regulated by guanine nucleotide exchange factors (GEFs), which facilitate the exchange of GDP for GTP, and GTPase-activating proteins (GAPs), which promote the hydrolysis of GTP back to GDP, thereby inactivating Ras. Once activated, RAS proteins trigger a downstream signaling cascade involving the RAF family of serine/threonine kinases, comprising A-RAF, B-RAF, and RAF-1 (also known as C-RAF), which translocate to the plasma membrane and activate one another. This activation subsequently leads to phosphorylation and activation of mitogen-activated protein kinase kinase (MEK) and extracellular signal-regulated kinase (ERK), culminating in changes to gene expression that govern cellular proliferation, differentiation, and survival. Mutations within this signaling pathway are frequently observed in human cancers. Mutations in KRAS (Kirsten rat sarcoma viral oncogene homolog) are particularly prevalent in pancreatic, colorectal, and non-small cell lung cancers [12]. In addition, BRAF (v-raf murine sarcoma viral oncogene homolog B1) mutations are found in approximately 8 % of all cancers and are especially common in melanoma. Although less frequent, mutations in MEK (MAP2K1/2) and ERK (MAPK1/3) have also been identified in certain malignancies. The B-Raf/Raf-1 pair typically drives cancer-related Ras signaling, with the V600 BRAF mutation being particularly significant as it can function independently of Ras. While KRAS was once considered impossible to target, new drugs can now bind to specific mutations like KRASG12C. Two such compounds, AMG510 and MRTX849, are currently under clinical evaluation. For BRAFV600E/K mutations, three approved drugs (dabrafenib, encorafenib, and vemurafenib) are available. However, these drugs can paradoxically activate MAPK signaling in cells without BRAFV600E mutations by promoting Raf-1 and B-Raf interaction. To address this, newer type-II RAF inhibitors were developed that can block both normal and mutant BRAF activity [13]. These “paradox breakers” show promise in treating resistant cancers, particularly when combined with other therapies. While earlier type-II RAF inhibitors had limited success, newer versions like AZ628, TAK632, and LXH254 show improved effectiveness across various cancer types. Resistance to Raf inhibitors often develops through MAPK pathway alterations or PI3K pathway activation. Combined targeting of both pathways shows promise but faces toxicity challenges. An alternative approach using lower doses of multiple drugs targeting insulin-like growth factor receptor (IGFR), KRAS, and mTOR has shown better tolerability. For treating BRAFV600E/K mutations in melanoma and lung cancer, three MEK1/2 inhibitors are available. Newer options include dual Raf/MEK inhibitors like RO5126766, currently being tested in various cancers. Since MEK inhibitor resistance often involves ERK1/2 reactivation, new ERK inhibitors are being developed as potential solutions. This evolving understanding of cellular signaling continues to yield new therapeutic strategies, though challenges remain in managing drug resistance and toxicity [14], [15].

#### Cyclin-dependent kinases (CDKs)

The cell cycle is regulated by CDKs, which form complexes with cyclins. These kinases are controlled by several inhibitory proteins, including p16INK4A, p15INK4B, p18INK4C, and p19INK4D. When cells receive growth signals, CDK4/6 joins with cyclin D1 to modify the retinoblastoma protein (RB) through phosphorylation. This modification disrupts RB's ability to suppress cell division by freeing the E2F transcription factor. The CDK4/6 complex also enhances forkhead box M1 (FOXM1)-driven gene expression, which helps control cell division and prevent cellular aging. In breast cancer treatment, drugs that block CDK4/6 have shown significant success. Three such inhibitors are now approved: abemaciclib (which can be used alone in certain cases), and ribociclib and palbociclib (typically used with other treatments) [16]. These drugs may also influence immune responses, suggesting potential combinations with immunotherapy. CDK4/6 inhibitors have proven effective in overcoming resistance to various cancer treatments. Research has shown they can restore sensitivity to hormone therapy and targeted treatments in breast cancer. For instance, combining these inhibitors with PI3K blockers has shown promising results in resistant breast cancers, particularly when high levels of phosphorylated RB are present. Studies have demonstrated that CDK4/6 inhibitors can also enhance the response of human epidermal growth factor receptor 2-positive (HER2-positive) breast cancers to trastuzumab. Similar therapeutic benefits have been observed with other targeted agents, including those directed against epidermal growth factor receptor (EGFR), mesenchymal-epithelial transition factor (MET)/tropomyosin receptor kinase (TRK), and MEK. However, cancer cells can develop resistance to CDK4/6 inhibitors through various mechanisms. These include changes in cell cycle regulators and activation of alternative growth pathways. Specific genetic changes in genes like TP53, AKT1, RAS, and others can lead to treatment resistance [17]. Additionally, amplification of specific genes such as fibroblast growth factor receptor 1 and 2 (FGFR1/2) and erb-b2 receptor tyrosine kinase 2 (ERBB2), along with alterations in multiple cellular signaling pathways, can contribute to the development of therapeutic resistance. Recent research has identified loss of PTEN as another mechanism of resistance to CDK4/6 inhibitors in breast cancer. Laboratory studies showed that removing PTEN made cells resistant to these drugs, but this resistance could be overcome by adding AKT inhibitors. This finding suggests that combining CDK4/6 inhibitors with AKT inhibitors might be effective in treating PTEN-deficient cancers. These insights continue to shape our understanding of how to best use CDK4/6 inhibitors and develop strategies to overcome treatment resistance [18].

#### Genomic instability-related signaling

Cell division involves careful replication of DNA, which is monitored by checkpoint systems that protect genetic information integrity. When DNA is damaged, cells activate various repair mechanisms. These include base-excision repair (BER) and nucleotide-excision repair (NER) for fixing single base-pair issues, while homologous recombination (HR) and non-homologous end joining (NHEJ) handle double-strand breaks. During cell division, mitotic checkpoints ensure proper chromosome distribution. If these repair systems fail, cells typically activate backup safety measures, leading to either cellular aging or death. Cancer develops when cells accumulate genetic instability, often due to the loss of tumor suppressors or activation of cancer-promoting genes [19]. Many cancers show mutations in genes responsible for DNA repair (such as BRCA1/2, partner and localizer of BRCA2 (PALB2), and RAD51 homolog C (RAD51C)) or those that guard genome stability (e.g., TP53, Ataxia Telangiectasia Mutated (ATM), and checkpoint kinase 2 (CHEK2). Despite having damaged DNA repair systems, cancer cells continue dividing rapidly in response to growth signals. Scientists have learned to use this vulnerability by developing treatments that target DNA repair pathways, pushing cancer cells toward self-destruction. While traditional chemotherapy drugs like topotecan, etoposide, cyclophosphamide, and cisplatin have been used to damage cancer cell DNA, newer treatments specifically targeting DNA repair pathways are still being developed and are not yet widely available [20].

#### PARP

Cells can repair DNA double-strand breaks (DSB) using two main methods: the accurate HR or the less precise NHEJ. In cells with functioning BRCA genes, HR is the preferred repair method during S/G2 phase. This process involves RAD51 recombinase using sister chromatid sequences as templates, supported by BRCA1/BARD1 and BRCA2/PALB2 complexes. In contrast, NHEJ relies on proteins like Ku70/80 and DNA-PKc to join broken DNA ends, but often introduces errors. When HR is impaired, such as in BRCA-deficient cells, cells depend more on PARylation and NHEJ [21]. BRCA mutations significantly increase breast and ovarian cancer risks, with BRCA2 mutations also raising prostate cancer risk. PARylation serves as an early DNA damage signal. PARP1 and PARP2 enzymes create PAR chains at damage sites to stabilize replication and recruit repair factors. PARP inhibitors (PARPi) block this process, leading to DNA damage accumulation and cell death in HR-deficient tumors. Several PARPi are now approved for treating various cancers, including certain breast, ovarian, and prostate cancers. For HR-proficient cancers that don't respond to PARPi alone, researchers have developed combination approaches. These include combining PARPi with PI3K inhibitors, which reduce BRCA1/2 expression, or with enhancer of zeste homolog 2 (EZH2) inhibitors, which suppress NHEJ activity [22]. Both strategies have shown promise in clinical trials. Resistance to PARPi typically occurs through HR pathway reactivation, often via BRCA function restoration through various mechanisms. To address this, researchers are exploring combinations with other checkpoint inhibitors targeting ataxia-telangiectasia mutated (ATM), ataxia-telangiectasia and Rad3-related protein (ATR), checkpoint kinase 1 (CHK1), aurora kinase A (AURKA), polo-like kinase (PLK), and dual-specificity protein kinase TTK (also known as monopolar spindle 1 kinase, Mps1) pathways. Another approach involves targeting poly (ADP-ribose) glycohydrolase (PARG), an enzyme that removes PAR chains after DNA repair. PARG inhibition causes PAR chain buildup, leading to replication problems and cell death. Recent research shows PARG inhibitors work effectively with G2/M checkpoint inhibitors, potentially offering a new treatment strategy for both homologous recombination (HR)-proficient and HR-deficient cancers [23].

#### p53 and p21

The tumor suppressor gene TP53, often called the genome's guardian, is the most commonly mutated gene in cancer. In normal cells, the levels of the p53 protein are tightly regulated by mouse double minute 2 homolog (MDM2), which targets it for degradation. However, under conditions of cellular stress, p53 accumulates and becomes activated. As a transcription factor, p53 regulates the expression of several critical genes, such as cyclin-dependent kinase inhibitor 1A (CDKN1A, also known as p21), which halts the cell cycle and promotes cellular senescence, and pro-apoptotic genes like BCL2-associated X protein (BAX) and p53 upregulated modulator of apoptosis (PUMA), which initiate programmed cell death (apoptosis). Researchers have developed drugs like nutlins to increase p53 levels by blocking MDM2 interaction, showing promising results in cancers with normal p53. Scientists have also explored ways to exploit p53′s function through synthetic lethality. For example, inhibition of ataxia telangiectasia and Rad3-related protein (ATR), a kinase essential for maintaining genomic integrity under replication stress, creates a dependence on p53-mediated DNA damage responses. Consequently, TP53-deficient tumors exhibit heightened sensitivity to ATR inhibitors. For example, when ATR (a protein that helps maintain genetic stability) is blocked, cells become dependent on p53 for DNA damage response. This makes p53-deficient cancers particularly vulnerable to ATR inhibitors. Additionally, p53 influences DNA repair pathway choice through p53-binding protein 1 (53BP1), making p53-mutant cells potentially more sensitive to treatments targeting HR [24]. The relationship between p21 (a protein controlled by p53) and cancer is complex. While drugs that increase p21 levels can kill cancer cells or stop their growth, recent research has revealed that high p21 levels can sometimes promote cancer progression. Scientists have found actively dividing cancer cells with high p21 levels in various tumor types. In p53-deficient cancers, sustained high p21 levels can lead to genomic instability and more aggressive cancer behavior by affecting DNA replication and repair mechanisms. These findings suggest new treatment strategies, such as targeting the Rad52 protein in p53-deficient cancers with high p21 levels. They also raise concerns about using certain treatments, like dexamethasone, which increase p21 levels independently of p53, in patients with p53-deficient cancers. The research highlights how both p53 mutations and elevated p21 levels can promote cancer development in specific contexts, emphasizing the need for careful consideration of treatment approaches based on a tumor's molecular profile [25] (Table 1).

**Table 1:** Key signaling pathways, components, and therapeutic resistance mechanisms in cancer.

Signaling pathway	Key components	Cancer types	Therapeutic approaches	Resistance mechanisms	References
PI3K/AKT/mTOR	PI3K, AKT, mTOR, PTEN	Breast, Endometrial, Prostate, Brain	− Pan-PI3K inhibitors (Buparlisib, Pictilisib)	−mTOR reactivation	[26], [27], [28], [29], [30], [31], [32], [33], [34], [35], [36], [37], [38]
			− Isoform-specific inhibitors (Idelalisib, Alpelisib)	−Alternative pathway activation	
			− AKT inhibitors (Ipatasertib)	− PTEN loss	

Ras/MAPK	RAS, RAF, MEK, ERK	Pancreatic, Colorectal, Lung, Melanoma	− KRAS G12C inhibitors (AMG510, MRTX849)	− KRAS/NRAS mutations	[39], [40], [41], [42], [43], [44], [45], [46], [47], [48], [49], [50], [51], [52]
			− B-RAF inhibitors (Dabrafenib, Vemurafenib)	− BRAF amplification	
			− MEK inhibitors (Binimetinib, Trametinib)	− MEK1/2 amplification	

CDK4/6	CDK4/6, Cyclin D1, RB	Breast Cancer (primarily)	− CDK4/6 inhibitors (Abemaciclib, Ribociclib, Palbociclib)	− RB loss	[53], [54], [55], [56], [57], [58], [59], [60], [61], [62], [63], [64], [65], [66], [67], [68], [69], [70], [71]
				− TP53 mutations	
				− PTEN loss	
				− FGFR1/2 activation	

DNA Repair	BRCA1/2, PARP, ATM/ATR	Breast, Ovarian, Prostate	− PARP inhibitors (Olaparib, Niraparib)	− BRCA reversion mutations	[72], [73], [74], [75], [76], [77], [78], [79], [80], [81], [82], [83], [84], [85]
				− HR pathway reactivation	
			− ATM/ATR inhibitors		
			− Checkpoint inhibitors		

p53/p21	p53, MDM2, p21	Multiple cancer types	− MDM2 inhibitors (Nutlins)	− p53 mutations	[86], [87], [88], [89], [90], [91], [92], [93], [94], [95], [96], [97], [98], [99], [100], [101]
				− p21 dysregulation	
			− Synthetic lethal approaches	− Alternative pathway activation	

## Current state of medical therapies in urological cancers

The treatment landscape for metastatic urological cancers has traditionally relied on specific approaches: tyrosine kinase inhibitors for renal cell cancer (RCC), cytotoxic chemotherapy for urothelial cancer (UC), and androgen deprivation therapy for prostate cancer (PCa). While these remain important, the field is experiencing significant changes with the introduction of immune checkpoint inhibitors (ICIs) that target PD-1, PD-L1/2, and cytotoxic T-lymphocyte associated protein 4 (CTLA-4). Recent additions include olaparib for treating specific mutations in castration-resistant prostate cancer, marking substantial progress in treatment options [102]. The treatment protocols for urological cancers continue to evolve rapidly. In RCC, five combination therapies involving immunotherapy drugs have gained approval, with research now focusing on post-surgery treatment. For invasive urothelial cancer, several clinical trials are exploring pre- and post-surgical treatments. While the MVAC chemotherapy regimen has been the standard for metastatic UC since 1985, ongoing trials are investigating ICI combinations that could establish new first-line treatments [103]. Prostate cancer research includes notable trials like ARASENS, which examines combination therapy for metastatic hormone-sensitive disease, studying various drug combinations for castration-resistant cases [104]. An emerging focus is genomic-based treatment approaches. Traditional tissue sampling for genetic analysis faces challenges, with 30 % of biopsies proving inadequate in the PROfound trial. Liquid biopsy technology, showing high concordance with tissue sampling, offers a promising alternative.

The rising global incidence and economic burden of prostate cancer necessitate a shift from reactive treatment to predictive, preventive, and personalized medicine (PPPM). Recent studies highlight the importance of cost-effective prevention strategies, including early risk assessment to identify individuals at high risk of aggressive cancer, advanced screening programs to detect prostate cancer at sub-optimal health stages for timely intervention, and phytochemical-based prevention using plant-derived compounds to enhance treatment efficacy and mitigate side effects. Personalized prevention integrates molecular phenotyping and predictive diagnostics to tailor interventions, with phytochemicals like silibinin playing a role in improving cancer management, particularly in vulnerable populations. A multi-professional approach is crucial for implementation, encompassing three levels of care: primary care, which focuses on early risk assessment and prevention; secondary care, which aims to prevent metastatic progression; and tertiary care, which involves personalized management of advanced disease. This comprehensive strategy is designed to reduce prostate cancer incidence and treatment costs while improving patient outcomes [105], [106].

Phenotyping is vital in prostate cancer treatment, enabling personalized care by analyzing genetic, clinical, and molecular traits. It helps distinguish between indolent and aggressive cases, supports targeted therapies, and aids in early detection through liquid biopsy. With rising metastatic cases in younger men, phenotyping refines prevention strategies and optimizes healthcare resources. Its integration into predictive and personalized medicine enhances precision, reduces costs, and improves patient outcomes [106], [107].

Nano-drug formulations are revolutionizing cancer theranostics by enhancing both diagnosis and treatment. These formulations leverage nanoparticles to improve drug delivery, targeting, and therapeutic efficacy while minimizing side effects. Key insights on nano-drug formulations in cancer theranostics include precision targeting, where nanomedicines can be engineered to selectively target cancer cells, reducing damage to healthy tissues [108].

Drug repurposing is a cost-effective strategy that finds new therapeutic uses for existing drugs with known safety profiles. Since these drugs have already undergone initial testing, repurposing accelerates development, reduces costs, and minimizes failure risks compared to creating new drugs from scratch. Using off-patent or generic drugs makes therapies more affordable, while known safety data allows clinical trials to focus on efficacy, shortening timelines. Repurposing is particularly beneficial for rare diseases with limited treatment options, and there have been notable successes such as hydroxychloroquine being repurposed from malaria treatment to lupus therapy, sildenafil transitioning from heart conditions to erectile dysfunction treatment, and thalidomide evolving from a sedative to a cancer and inflammatory disease therapy. Clinical trials for repurposed drugs often benefit from accelerated timelines as safety profiles are already established, allowing them to bypass early-phase safety testing [109]. However, these trials may face challenges such as small sample sizes, participant heterogeneity, and difficulties in demonstrating efficacy for new indications, particularly in complex diseases like hematological malignancies. Some repurposed drugs have shown limited success in trials for certain conditions, highlighting the need for rigorous evaluation. Advances in computational modeling, artificial intelligence, and data analytics are improving drug selection and trial design, increasing the likelihood of successful repurposing. Regulatory agencies like the FDA and European Medicines Agency (EMA) provide streamlined pathways and incentives to facilitate approval, especially for rare or unmet medical needs. In conclusion, drug repurposing enhances accessibility and affordability in healthcare, offering promising solutions for various medical challenges while leveraging existing treatments [110].

Mitochondrial health plays a crucial yet underexplored role in the progression and treatment of urological cancers, including bladder and prostate cancer. Mitochondrial dysfunction contributes to therapy resistance by impairing apoptosis, allowing cancer cells to survive and proliferate. Alterations in mitochondrial DNA and oxidative phosphorylation (OXPHOS) are linked to cancer progression and treatment evasion. Therapeutic strategies targeting mitochondria, such as dichloroacetate (DCA) and metformin, restore mitochondrial function and induce apoptosis by modulating metabolic pathways. Vitamin K2 triggers metabolic stress in UC cells, leading to autophagic cell death, while emerging mitochondria-targeted therapies, including antisense oligodeoxynucleotides and heat shock protein modulators, aim to restore apoptosis and overcome resistance. In prostate cancer, androgen deprivation therapy (ADT) negatively impacts mitochondrial health, causing metabolic disruption, oxidative stress, and systemic toxicities, including neurocognitive decline. Mitochondria-targeted agents like Mito-Lonidamine (Mito-LND) enhance radiotherapy response by inhibiting mitochondrial respiration and reducing tumor hypoxia. Future advancements include mitochondrial transplantation, a novel strategy that may increase sensitivity to chemotherapy in prostate cancer. Further research into mitochondrial biology in urological cancers could lead to personalized therapies based on mitochondrial fitness and metabolic profiles. Integrating mitochondrial biomarkers and targeted interventions holds significant potential for improving treatment outcomes and refining clinical strategies [111] (Table 2).

**Table 2:** Overview of traditional and novel therapies, ongoing clinical trials, and future directions.

Cancer type	Traditional therapies	Novel therapies	Ongoing clinical trials	Future directions	References
Renal cell cancer (RCC)	Tyrosine kinase inhibitors (TKIs)	Combination therapies	Focus on adjuvant therapy after radical nephrectomy	Shifting towards adjuvant treatments	[103], [120], [121]
		− Nivolumab + Ipilimumab			
		− Pembrolizumab + Axitinib			
		− Avelumab + Axitinib			
		− Pembrolizumab + Lenvatinib			
		− Nivolumab + Cabozantinib			

Urothelial cancer (UC)	Cisplatin-based chemotherapy (MVAC, GC regimen)	− Immune Checkpoint Inhibitors (ICIs)	− CheckMate274	New first-line regimens with ICIs compared to GC	[114], [115], [116], [117], [118], [119], [122]
			− AMBASSADOR < br>- Keynote-905/EV-303		
			− IMvigor010		
		− Enfortumab vedotin (ADC)			

Prostate cancer (PCa)	Androgen Deprivation Therapy (ADT)	− PARP inhibitor (Olaparib)	− ARASENS trial (ADT + docetaxel + darolutamide)	− 177Lu-PSMA-617 radioligand therapy	[102], [112], [113], [123], [124], [125], [126], [127], [128]
		− Combination therapies			
			− KeyLynk-010 (ADT + olaparib + pembrolizumab)		
				− Genome information-based therapy	
				− Liquid biopsy advances	

## Emerging targeted therapies and clinical trials for urologic neoplasms based on signaling pathways

### Bladder cancer

**Fig. 2:**
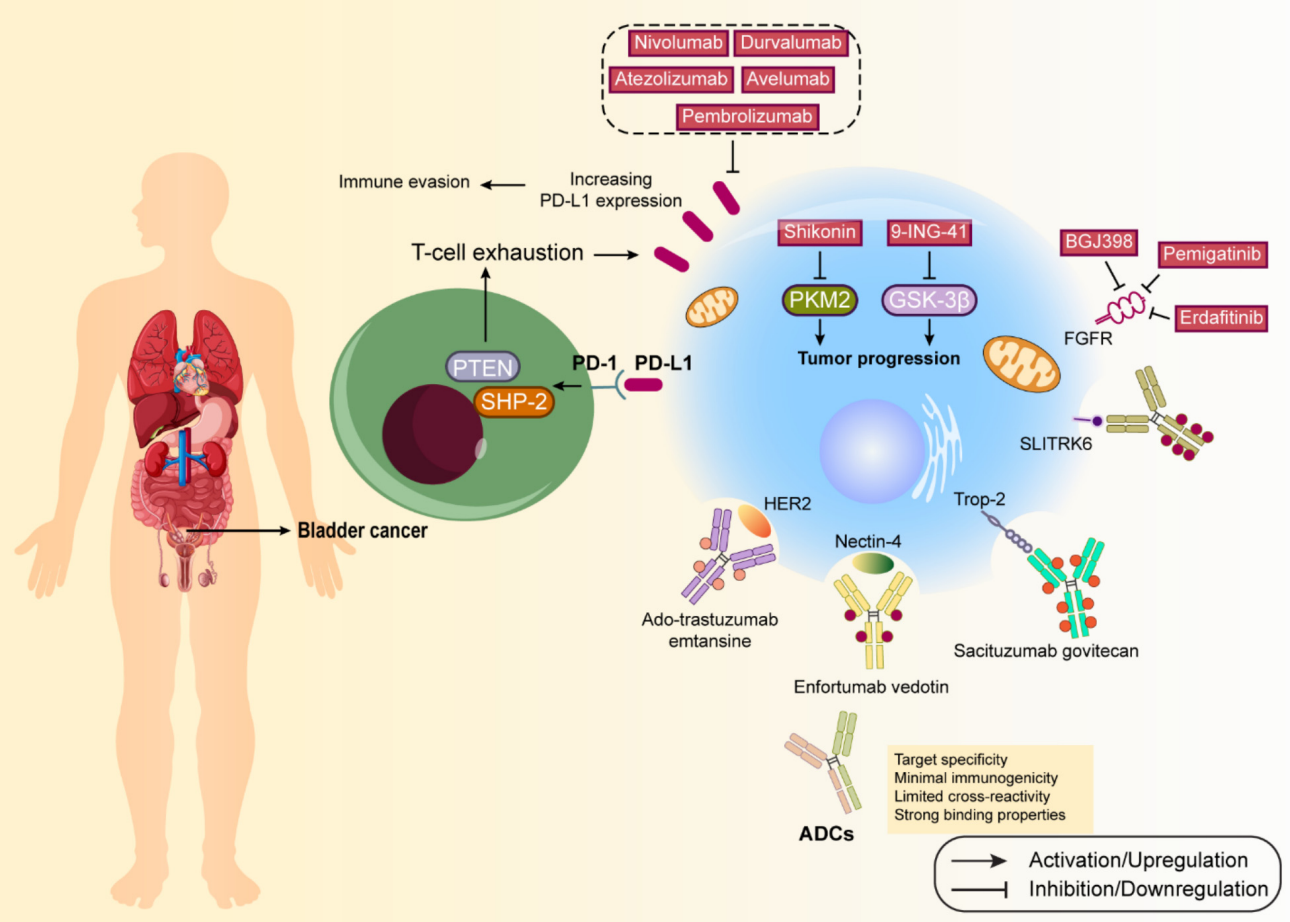
Targeted therapies and clinical trials for bladder cancer.

Bladder cancer represents the predominant form of urinary system malignancies, comprising 90–95 % of urothelial carcinomas, which includes cancers of the bladder, upper urinary tract, and proximal urethra. Projections indicate approximately 81,180 new bladder cancer cases will emerge in the USA by 2022. Disease classification follows either the tumor-node-metastasis system, measuring invasion depth, or WHO criteria, which categorizes tumors as high- or low-grade based on cellular features. Tumors are further categorized as nonmuscle-invasive (NMIBC) or muscle-invasive (MIBC) based on their penetration depth, with distinct progression patterns between these types. Immunotherapy has emerged as a powerful treatment approach for bladder cancer, utilizing both BCG for early stages and immune checkpoint inhibitors for advanced disease. BCG, originally a tuberculosis vaccine, became the first approved immunotherapy for cancer in 1990, following William B. Coley's pioneering work with microbial products in cancer treatment [129]. Current guidelines recommend BCG treatment with a six-week induction phase followed by maintenance courses at specific intervals. The mechanism of BCG's anti-tumor activity involves multiple steps. After installation, BCG binds to urothelial cells through specific proteins and gets internalized by various immune cells. It triggers direct cancer cell death through cytokine release while activating both innate and adaptive immune responses. The treatment recruits various immune cells including lymphocytes, macrophages, neutrophils, and natural killer cells to the bladder tissue. BCG therapy also initiates adaptive immunity primarily through T cell responses. Dendritic cells and urothelial cells present BCG antigens, activating CD4+ T cells which predominantly develop into T helper 1 (TH1) cells. The balance between TH1 and TH2 responses significantly influences treatment success, with TH1-dominated responses (characterized by specific cytokines like IL-2 and IFNγ) associated with better outcomes, while TH2-dominated responses often indicate treatment resistance [130], [131]. More details on targeted therapies against bladder cancer are provided in the following, as well as Fig. 2.

#### Immune checkpoint inhibitors (ICIs)

The successful 40-year history of BCG immunotherapy in NMIBC treatment demonstrates bladder cancer's susceptibility to immune-based interventions. This field has seen renewed interest with the development of ICIs. Key immune checkpoint molecules include PD-1, its ligands (PD-L1 and PD-L2), and CTLA-4. PD-1, found on activated immune cells, is a transmembrane glycoprotein that, when bound to PD-L1, triggers a signaling cascade involving SHP-2 and PTEN, ultimately leading to T-cell exhaustion. This mechanism allows cancer cells to evade immune detection by increasing PD-L1 expression. CTLA-4, expressed primarily on activated T cells, interacts with B7-1 (CD80) and B7-2 (CD86) molecules on antigen-presenting cells [132], [133]. This interaction initiates a phosphorylation cascade that maintains CTLA-4′s surface expression and suppresses T cell responses through protein phosphatase 2A (PP2A) activation. Five ICIs have received regulatory approval for metastatic bladder cancer treatment. Pembrolizumab and atezolizumab serve as first-line therapies for patients unsuitable for cisplatin-based treatments who are PD-L1 positive. These treatments have shown promising results in clinical trials, though atezolizumab was later withdrawn as a second-line treatment due to mixed outcomes. Additional approved second-line treatments include nivolumab, avelumab, and durvalumab, each demonstrating varying degrees of effectiveness. Nivolumab showed a 19.6 % objective response rate regardless of PD-L1 expression, while avelumab and durvalumab demonstrated antitumor activity with acceptable safety profiles. However, durvalumab was later withdrawn as a second-line treatment. Anti-CTLA-4 antibodies (ipilimumab and tremelimumab) remain unapproved for bladder cancer due to inconsistent trial results. Some studies suggest potential benefits of combination therapy, particularly nivolumab with ipilimumab, showing sustained antitumor activity with manageable side effects. Recent analyses indicate that combination immunotherapy may offer better survival outcomes with lower toxicity compared to combination chemotherapy, though further research is needed to confirm these findings [134], [135].

#### ADCs

The successful integration of dendritic cell-based treatments with monoclonal antibody-linked cytotoxins represents a significant advancement in bladder cancer therapy, particularly for patients who have undergone prior immunotherapy and chemotherapy. Antibody-drug conjugates (ADCs) combine targeting antibodies, cytotoxic payloads, and connecting linkers into a single therapeutic entity, offering unique advantages by targeting surface proteins regardless of their cellular growth functions. Optimal ADC effectiveness relies on target specificity, minimal immunogenicity, limited cross-reactivity, and strong binding properties. IgG1, the predominant antibody subtype used in ADC development, offers robust immune-stimulating capabilities. These compounds function as precision-guided therapeutics, entering cancer cells through receptor-mediated endocytosis before releasing their payload to induce cell death through microtubule or DNA disruption, with some payloads capable of affecting nearby cells through bystander effects[136]. Two ADCs have achieved regulatory approval for bladder cancer treatment. Enfortumab vedotin (EV), targeting Nectin-4, has demonstrated superior survival outcomes compared to chemotherapy and shows particular promise when combined with pembrolizumab for cisplatin-ineligible patients. Sacituzumab govitecan (SG), targeting Trop-2, has proven effective in advanced urothelial cancer patients who previously received platinum-based therapy and immunotherapy. The field continues to expand with several promising candidates under investigation. HER2-targeting compounds, including ado-trastuzumab emtansine and disitamab vedotin, have shown encouraging early results. Sirtratumab vedotin, targeting SLITRK6, demonstrates effectiveness in initial trials. Alternative approaches, such as peptide-drug conjugates like oportuzumab monatox and tissue factor-targeting compounds like tisotumab vedotin, are also showing promise in clinical development. These emerging therapies mark a significant step forward in targeted treatment options for bladder cancer, offering new possibilities for patients who have exhausted conventional treatment approaches. The continued development and refinement of these targeted therapies suggest an increasingly optimistic outlook for bladder cancer treatment [137], [138].

#### Tyrosine kinase inhibitors (TKIs)

TKIs have emerged as crucial therapeutic agents in cancer treatment. A significant milestone was reached in 2019 with the approval of erdafitinib, the first oral FGFR3-targeting molecule, for treating bladder cancer with specific FGFR2/3 mutations. This drug serves as either a third-line treatment or a second-line option after platinum-based chemotherapy fails. Clinical research has demonstrated erdafitinib's effectiveness in treating metastatic or inoperable urothelial carcinoma [139], with studies showing a 40 % objective response rate. The drug's dosing can be optimized by monitoring phosphate levels, and long-term safety data has been encouraging. Other FGFR inhibitors have shown promise. Pemigatinib, which selectively targets FGFR 1–3, has demonstrated effectiveness in various solid tumors, achieving a duration of response averaging 7.3 months. BGJ398, another FGFR1-3 inhibitor, has shown positive results with a disease control rate of 64.2 % in patients with FGFR3-modified metastatic urothelial cancer. Rogaratinib, which inhibits all FGFR types, has shown potential in advanced cases, particularly in patients previously treated with platinum therapy. Studies indicate a 20.7 % response rate and median survival of 8.3 months, with manageable adverse effects [140]. The EGFR pathway has become a central focus in targeted therapy research. Recent studies have uncovered complex interactions between EGFR and various molecular components, including long non-coding RNAs like EGFR antisense RNA 1 (EGFR-AS1), which influences cancer progression by stabilizing EGFR mRNA. Related proteins such as Annexin A1 and thyroid hormone receptor interactor 13 (TRIP13) have been identified as significant factors in bladder cancer development through EGFR pathway interaction. Research has also revealed new signaling networks, particularly the EGF-SHCBP1-RACGAP1-RAC1 pathway, which plays a crucial role in cancer cell migration. These discoveries are expanding our understanding of EGFR-targeted therapies. Various vascular endothelial growth factor receptor (VEGFR)-targeting TKIs have been studied in combination therapies. While nintedanib combined with standard chemotherapy proved safe, it didn't significantly improve outcomes. However, the combination of famitinib (targeting multiple receptors) with camrelizumab (anti-PD-1) showed promising results in advanced cases. Studies of other kinase inhibitors, including SRC inhibitors like bosutinib and dasatinib, continue to explore new therapeutic possibilities. Recent research has also highlighted the potential of targeting pathways involving focal adhesion kinase (FAK) and transient receptor potential melastatin 7 (TRPM7) in the treatment of bladder cancer [141].

#### Drug development against metabolic dysfunction-related signaling pathways

Cancer cells exhibit distinctive patterns in glucose metabolism. Recent research has identified several key metabolic regulators as potential therapeutic targets. In bladder cancer, multiple oncogenic factors, including glucose transporter 1 (GLUT1), c-Myc, and PI3K, enhance glycolytic activity. The metabolic pathway begins with increased glucose uptake through GLUT1, leading to elevated pyruvate production. This pyruvate follows two main paths: it either enters mitochondria for energy production through the tricarboxylic acid cycle, or it converts to lactic acid in the cytoplasm through lactate dehydrogenase (LDH) activity. Studies show that compounds like sulforaphane can modulate this process by affecting the AKT1/hexokinase 2 (HK2) pathway and pyruvate dehydrogenase (PDH) expression [142]. Bladder cancer cells show elevated levels of metabolic regulators such as 6-phosphofructo-2-kinase/fructose-2,6-biphosphatase 4 (PFKFB4) and pyruvate kinase M2 (PKM2) compared to normal cells. Therapeutic approaches targeting these molecules, such as using shikonin to inhibit PKM2, have shown promise in overcoming drug resistance. Mitochondrial function plays a crucial role in cancer metabolism. In bladder cancer, various mitochondrial proteins show abnormal expression patterns. Therapeutic strategies targeting mitochondrial metabolism have shown potential, including the use of specific inhibitors and photosensitizers. Metformin, traditionally used for diabetes, has emerged as a promising treatment option. It works by activating AMP-activated protein kinases and inhibiting mitochondrial complex 1 [143]. While laboratory studies show promising results, clinical trials are ongoing to validate its effectiveness in bladder cancer treatment. Glycogen metabolism also influences cancer development. Research has identified AGL as a potential tumor suppressor, with low expression correlating with poor survival rates. Glycogen synthase kinase 3β (GSK-3β) inhibitors, such as Elraglusib (9-ING-41), are being evaluated in clinical trials, showing enhanced effectiveness when combined with conventional chemotherapy. Lipid metabolism's role in bladder cancer has gained attention recently. Bioinformatics studies have identified several lipid metabolism-related genes as prognostic markers. Proteins like clusterin and mex-3 RNA binding family member C (MEX3C) have been shown to influence tumor growth through lipid metabolism pathways. These findings suggest that targeting lipid metabolism could offer new therapeutic approaches for bladder cancer treatment. The interaction between various metabolic pathways in cancer cells presents multiple potential therapeutic targets. Ongoing research continues to uncover new metabolic regulators and their roles in bladder cancer development and treatment [138].

#### Targeted therapies against bladder cancer stem cells-related signaling pathways

Research has shown that the Wnt/β-Catenin signaling pathway significantly influences tumor development and resistance to chemotherapy. This pathway is particularly important for maintaining cancer stem cells (CSCs). Studies demonstrate that cells resistant to paclitaxel show heightened Wnt/β-Catenin activity and increased stem cell characteristics. Manipulating β-Catenin levels affects drug sensitivity, with higher levels reducing effectiveness and lower levels restoring drug sensitivity. The Hippo-Yes-associated protein (YAP) pathway's dysfunction contributes to cancer development. YAP, a crucial pathway component, shows elevated expression in bladder cancer stem cells and regulates stemness through ALDH1A1 gene expression. It also influences drug resistance by controlling sex determining region Y-Box 2 (SOX2) expression and promoting autophagy, which helps CSCs survive treatment and maintain their characteristics. Signal transducer and activator of transcription 3 (STAT3) serves as a key regulator of CSCs by controlling epithelial-mesenchymal transformation. It becomes particularly active in radiation-resistant cells, enhancing their survival and invasive capabilities[144]. The IL-6/IL6R/STAT3 pathway maintains stem cell properties in bladder cancer, with drugs like tozumab showing promise in targeting this mechanism. Cyclooxygenase 2 (COX2), exclusively expressed in bladder cancer cells, participates in tumor regeneration through prostaglandin E2 (PGE2) signaling. This pathway affects SRY-box transcription factor 2 (SOX2) expression by modulating let-7, influencing stem cell properties and drug resistance. Treatments targeting this pathway, including metformin and celecoxib, show potential in combating cancer progression. Aldehyde dehydrogenase (ALDH) enzymes play vital roles in cellular detoxification and are associated with drug resistance in CSCs. High ALDH1A1 levels correlate with increased cancer cell proliferation and influence treatment outcomes through the retinoic acid pathway [134]. However, developing specific ALDH inhibitors remains challenging due to potential toxicity concerns. The β-arrestin (ARRB) protein family exhibits opposing roles in regulating stem cell-like characteristics in bladder cancer. β-arrestin 1 (ARRB1) enhances cancer stem cell properties and facilitates metabolic reprogramming, whereas β-arrestin 2 (ARRB2), also known as arrestin β2, acts as a suppressor of these traits. Although directly targeting β-arrestins remains a significant challenge, small-molecule compounds such as barbadin have shown therapeutic potential by modulating the interactions between β-arrestins and their associated signaling proteins. The Sonic Hedgehog (SHH) pathway influences cancer initiation and progression through its effects on stem cell renewal. TGF-β1 activates this pathway, promoting cancer cell migration and invasion. While compounds like cyclopamine can inhibit SHH signaling, more research is needed to fully understand how to effectively target this pathway in bladder cancer treatment. These various signaling pathways present multiple potential therapeutic targets, though developing effective treatments requires careful consideration of their complex interactions and potential side effects [141], [145].

### Prostate cancer

Worldwide, prostate cancer impacts a substantial male population. Treatment options have evolved significantly thanks to enhanced knowledge of genomic profiles and biological mechanisms. The therapeutic landscape now includes several innovative drug classes: enhanced androgen receptor (AR) pathway inhibitors (like abiraterone and enzalutamide), treatments targeting bone metastases (including radium-223 chloride), and PARP inhibitors such as olaparib. Clinical trials are exploring additional pathways involving CDK4/6, AKT, and WNT signaling. The emergence of prostate-specific membrane antigen (PSMA) −targeted therapies, particularly 177Lu-PSMA-617, shows promise in both diagnosis and treatment. While immune checkpoint inhibitors have shown limited overall effectiveness, they may benefit specific patient subgroups with MMR or CDK12 deficiencies [146].

The androgen signaling pathway is fundamental to prostate cancer development. Through the hypothalamic-pituitary–gonadal axis, androgen production is carefully regulated [147], [148]. When androgens bind to AR, it separates from heat shock proteins and moves to the nucleus, where it influences gene expression affecting both tumor growth and normal prostate development [149]. The groundbreaking work of Huggins and Hodges established androgen deprivation therapy (ADT) as a cornerstone treatment by demonstrating how surgical castration inhibits cancer progression. Modern prostate cancer treatment focuses on disrupting androgen signaling through various approaches: blocking gonadotropin-releasing hormone (GnRH) to prevent the production of luteinizing hormone (LH), inhibiting the enzyme cytochrome P450 17A1 (CYP17A1) to reduce androgen synthesis, or directly targeting the function of the AR [150].

AR, which consists of 919 amino acids and includes specific functional domains (N-terminal domain (NTD), DNA-binding domain (DBD), hinge region, and ligand-binding domain (LBD), is crucial in prostate cancer development and is present in most prostate cancer cases. Higher nuclear AR levels in bone metastases correlate with poorer outcomes. AR activation supports cancer cell growth through complex molecular mechanisms involving hormone binding, nuclear translocation, and regulation of target genes [151], [152], [153], [154].

Modern AR inhibitors have evolved beyond early treatments like bicalutamide. Enzalutamide, approved in 2012, demonstrates enhanced AR binding and has significantly extended survival rates in various prostate cancer stages. Apalutamide, introduced in 2018, shows even greater effectiveness, particularly in preventing metastasis in non-metastatic cases [155]. The emergence of AR mutations in about 60 % of metastatic castration-resistant cases led to the development of darolutamide, which uniquely targets mutated AR forms. A promising new approach involves AR protein degraders like ARV-110, which shows potential in overcoming treatment resistance. However, AR variants lacking the LBD remain a therapeutic challenge [156], [157], [158], [159], [160], [161], [162], [163].

GnRH, a key hypothalamic peptide, orchestrates testosterone production through the pituitary–gonadal axis. The development of GnRH agonists in the 1980s revolutionized treatment by suppressing testosterone to castration levels, though these drugs initially cause a temporary hormone surge [155]. To address this “flare-up” issue, GnRH antagonists were developed. While abarelix was discontinued due to adverse reactions, degarelix and the newer oral drug relugolix have proven more successful. However, these treatments can affect body composition and may raise cardiovascular concerns [155], [164], [165], [166], [167], [168].

**Fig. 3:**
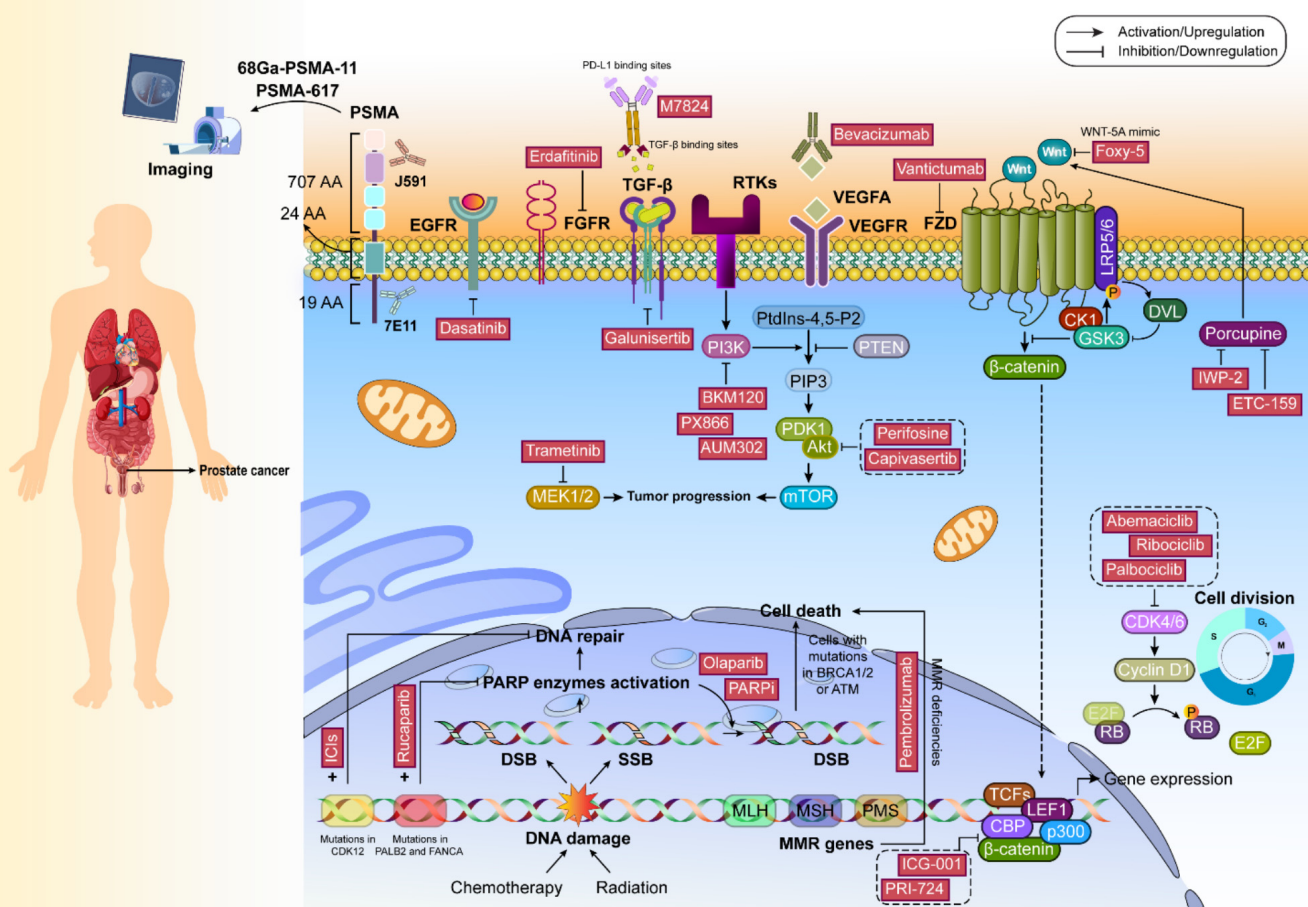
Targeted therapies and clinical trials for prostate cancer.

CYP17A1, essential for androgen synthesis, functions through dual enzymatic activities that produce testosterone precursors. Its role in intratumoral androgen production makes it a crucial target in castration-resistant cases [155]. Abiraterone, approved in 2011, blocks both CYP17A1 activities and has shown significant benefits in various patient groups. While it requires prednisone co-administration to manage side effects, newer drugs like orteronel and galeterone offer alternative approaches. Seviteronel represents an advancement by selectively targeting one enzyme activity without requiring prednisone supplementation. However, resistance development remains a challenge, as evidenced by increased CYP17A1 expression in treated tumors [169], [170], [171], [172], [173], [174]. This newer generation of treatments has shifted the therapeutic landscape, though challenges persist in managing resistance and optimizing treatment sequences. Ongoing research continues to refine these approaches and explore combination strategies to improve patient outcomes. Please refer to Fig. 3 for detailed information on prostate cancer targeted therapy.

### Targeting PSMA

#### PSMA and PSMA-targeted ligands

PSMA functions as a complex glycoprotein spanning the cell membrane. Its structure encompasses three distinct regions: a short internal segment of 19 amino acids, a membrane-spanning portion of 24 amino acids, and an extensive external region containing 707 amino acids. This protein exhibits both folate hydrolase and dipeptidase activities. While normal tissues show minimal PSMA presence, prostate cancer cells dramatically overexpress it − up to 1000-fold higher − making it an invaluable target for both diagnosis and treatment. Scientists have developed three distinct targeting approaches: antibodies, aptamers, and small molecules. The antibodies target either internal components (like 7E11) or external regions (such as J591). The humanized J591 antibody shows particular promise. Various aptamers, composed of nucleic acid sequences, can specifically recognize PSMA. Small-molecule compounds like PSMA-617 have become particularly useful for imaging and therapy. Early diagnostic efforts used ProstaScint, though its effectiveness was limited by only binding to internal PSMA regions. Modern techniques employ various radioactive tracers, with 68 Ga-PSMA-11 emerging as a leading option for PET imaging. Newer 18F-based agents offer improved characteristics, including enhanced precision and longer useful periods. Clinical studies demonstrate impressive accuracy, with some trials showing detection rates above 85 % for affected lymph nodes [175], [176], [177], [178], [179], [180], [181].

PSMA-targeted radiation treatment differs from traditional approaches by delivering radioactive compounds directly to cancer cells. These treatments use either beta-emitting isotopes (typically lutetium-177) or alpha-emitters (such as actinium-225). Recent studies suggest these methods may outperform conventional chemotherapy, leading to FDA approval of Lu-PSMA-617 in 2021. Scientists have also developed antibody-drug combinations targeting PSMA, such as MED13726 [185], [186]. While some early attempts faced challenges with stability or side effects, newer versions show promise. For instance, BIND-014 has demonstrated good tolerability in initial trials. The newest approach involves modifying immune cells to target PSMA. These engineered T-cells have evolved through several generations, with newer versions incorporating additional signaling components to enhance their effectiveness. While showing promise, this treatment requires careful monitoring for immune-related complications. All these approaches continue to evolve, with ongoing research focusing on optimizing patient selection and treatment timing. The field faces continuing challenges in managing treatment resistance and side effects while working to improve overall outcomes [182], [183], [184], [185], [186], [187], [188].

### Targeting DNA repair pathways

#### PARP function in DNA repair and synthetic lethality

The PARP enzyme family plays a crucial role in maintaining DNA integrity and regulating gene expression. These enzymes become activated when DNA damage occurs, coordinating repair processes through various mechanisms. DNA can be damaged in multiple ways − from medical treatments like chemotherapy and radiation, or from naturally occurring substances in cells. The body employs different repair strategies depending on whether single strands (SSB) or both strands (DSB) of DNA are broken [84], [189], [190], [191], [192].

PARP inhibition works through a principle called synthetic lethality, where combining two otherwise harmless genetic changes becomes fatal to cells. When PARP function is blocked, single-strand breaks accumulate and eventually develop into double-strand breaks. Normal cells can handle this situation, but cancer cells with mutations in repair genes like BRCA1/2 or ATM cannot survive this combined assault. This vulnerability is particularly relevant in advanced prostate cancer, where up to 30 % of cases show defects in DNA repair genes. The effectiveness of PARP inhibition may extend beyond this basic mechanism. Research suggests it might also work by disrupting other cellular processes and could be especially effective in cancers with certain genetic features, such as transmembrane protease serine 2–ETS-related gene (TMPRSS2-ERG) fusion or PTEN loss. Additionally, hormone therapy's effectiveness appears connected to DNA repair capabilities, and compromised repair systems make cancers more vulnerable to various treatments. Several PARP-blocking drugs have shown promising results in clinical studies. Olaparib received FDA approval in 2020 after demonstrating significant benefits, particularly in patients with specific genetic mutations. In clinical trials, 88 % of patients with certain DNA repair deficiencies responded to treatment, with BRCA1/2 mutation carriers showing notably improved survival rates. Rucaparib, another FDA-approved drug, has shown varying effectiveness depending on the specific genetic mutations present. While some patients with mutations in genes like partner and localizer of BRCA2 (PALB2) and Fanconi anemia complementation group A (FANCA) responded well, those with ATM alterations showed less improvement. Other drugs like talazoparib have demonstrated moderate success rates but require careful monitoring for side effects. Current research explores combining PARP inhibitors with other treatments. For instance, studies combining veliparib with hormone therapy showed improved results in patients with DNA repair deficiencies. However, identifying which patients will benefit most from these combination approaches remains challenging. This field continues to evolve as researchers develop new drugs like niraparib and pamiparib, currently under clinical evaluation. The challenge lies in determining the most effective treatment combinations and identifying reliable markers to predict treatment success [193], [194], [195].

### Targeting immune checkpoints

#### MMR defect and immunotherapy response

Cancer cells with defective mismatch repair (MMR) genes or microsatellite instability tend to trigger stronger immune responses, marked by increased immune cell infiltration. This enhanced immune response occurs because these defects lead to more mutations and novel protein fragments (neoantigens) that the immune system can recognize. These distinctive proteins appear on cell surfaces through MHC class I molecules, enabling immune cells to identify and attack abnormal cells. The DNA mismatch repair system relies on several key proteins-MutL homolog 1 (MLH1), MutS homolog 2 (MSH2), MSH6, and postmeiotic segregation increased 2 (PMS2) −which correct errors during DNA copying and recombination. In prostate cancer, roughly 3–5 % of cases show deficiencies in these repair proteins, resulting in increased mutation rates and unstable microsatellites. These changes typically correlate with higher levels of neoantigens and increased presence of tumor-fighting immune cells [196], [197], [198], [199], [200].

Scientists have tested various ICIs targeting different proteins like PD-L1, PD1, and CTLA4. While initial results were modest, researchers discovered that patients with MMR deficiencies often respond better to these treatments. This led to FDA approval of pembrolizumab for cancers, including prostate cancer, with specific MMR or microsatellite abnormalities. However, treatment success varies significantly. For instance, in one study, just over half of advanced prostate cancer patients with MMR defects showed significant improvement with pembrolizumab. The reasons why some patients don't respond remain unclear. This has led researchers to explore combination approaches, mixing ICIs with traditional chemotherapy, hormone treatments, or PARP inhibitors, with some promising early results. A significant discovery involves CDK12 mutations, present in about 10 % of advanced prostate cancer cases. CDK12, working with cyclin K, plays a vital role in DNA repair during gene expression. When CDK12 stops working properly, it causes specific genetic changes that increase protein variations and enhance immune responses. Early studies show that some patients with CDK12 mutations respond well to immune checkpoint therapy, leading to ongoing clinical trials testing combined immunotherapy approaches [201], [202], [203].

These findings highlight the complex relationship between DNA repair mechanisms and immune responses in cancer treatment, suggesting potential new therapeutic strategies based on specific genetic features of individual tumors.

### Targeting the cell cycle

#### CDK4/6 and cell cycle

Cell proliferation control is fundamental to preventing cancer development. The cell cycle consists of four distinct phases − G1, S, G2, and M − with critical checkpoints regulated by specific proteins, particularly CDKs. CDK4 and CDK6 are especially important in controlling the transition from G1 to S phase. These proteins become active by joining with cyclin D proteins in response to growth signals. The CDK4/6-cyclin D partnership leads to changes in the RB protein through phosphorylation. Normally, RB prevents cell division by binding to E2F transcription factors. When phosphorylated, RB releases E2F, allowing it to activate genes necessary for cell division. Beyond cell cycle regulation, CDK4/6 also influences cell specialization and metabolism [204], [205], [206].

Three CDK4/6-blocking drugs − palbociclib, ribociclib, and abemaciclib − are being tested in prostate cancer. Early studies combining hormone therapy with palbociclib showed similar effectiveness to hormone therapy alone, suggesting potential benefits from targeting both hormone signaling and cell cycle control. Ongoing research explores combinations with other treatments, including immunotherapy, as CDK4/6 inhibitors may enhance immune responses against tumors [207], [208], [209].

The p53 protein, often called the genome's guardian, plays a crucial role in preventing cancer development by triggering cell death or stopping cell division when DNA damage occurs. In prostate cancer, particularly aggressive forms, p53 mutations are common and often occur alongside RB alterations. These mutations can either disable p53′s protective functions or create new harmful effects. While some of these treatments show promise in other cancers, their effectiveness in prostate cancer requires more research. The FDA has fast-tracked certain p53-targeting drugs for specific cancers, and early trials show encouraging results. However, more clinical studies are needed to determine their effectiveness in prostate cancer treatment. These developments reflect ongoing efforts to understand and target key cellular control mechanisms in cancer treatment, with potential for more effective therapies in the future [210], [211], [212], [213], [214].

### Targeting the PI3K/AKT/mTOR signaling axis

#### PTEN/PI3K/AKT/mTOR signaling

About 20 % of initial prostate cancers and 35 % of advanced cases show PTEN protein inactivation. PTEN works as an enzyme that converts PIP3 to PIP2, effectively counteracting PI3K, which does the opposite conversion. When PTEN stops working, PIP3 builds up abnormally in cell membranes, triggering a chain reaction through 3-phosphoinositide-dependent protein kinase-1 (PDK1) and AKT. This activated pathway affects various cell functions through mTOR, including protein production, cell survival processes, growth, and energy use. The loss of PTEN function typically indicates a worse outlook in advanced prostate cancer due to increased PI3K/AKT/mTOR pathway activity. PI3K inhibitors like BKM120 and PX866 work by blocking all forms of PI3K's catalytic unit, particularly p110β, which is especially important in prostate cancer. While these drugs are generally well-tolerated, using them alone has shown limited success. AKT-targeting drugs come in two types: those that change AKT's shape (such as perifosine) and those that compete with ATP (like capivasertib). Some promising results have emerged, especially when combining these drugs with standard treatments. For instance, capivasertib plus docetaxel showed significant benefits in advanced cases. Proviral integration site for Moloney murine leukemia virus (PIM) proteins, which help maintain the PI3K/AKT/mTOR pathway, have emerged as another important target. These proteins are often overactive in prostate cancer and can make tumors resistant to various treatments. New drugs that simultaneously target PIM, PI3K, and mTOR, like AUM302, show promise and might cause fewer side effects than using multiple separate drugs. Despite these advances, effectively using these treatments in prostate cancer remains challenging. More research is needed to identify which patients will benefit most and to develop better combination strategies [215], [216], [217], [218], [219].

### Targeting WNT signaling

#### WNT/β-catenin signaling

The WNT/β-catenin signaling pathway becomes activated in late-stage prostate cancer, promoting tumor growth and drug resistance [220]. When WNT proteins bind to their cell surface receptors, they trigger pathways controlling cell differentiation and multiplication. Without WNT signals, β-catenin in the cytoplasm gets quickly broken down by a destruction complex containing adenomatous polyposis coli (APC), AXIN, casein kinase 1 (CK1), β-transducin repeat containing protein (β-TrCP), and GSK3 [221], [222], [223]. When WNT binds to Frizzled (FZD) receptors [224] and LRP5/6 co-receptors, it leads to LRP5/6 phosphorylation by CK1 and GSK3. This activates Dishevelled (DVL) protein, which brings the destruction complex to the cell membrane. As a result, GSK3 is inhibited, preventing β-catenin breakdown and allowing it to accumulate. β-catenin subsequently translocates into the nucleus, where it associates with T-cell factor/lymphoid enhancer-binding factor 1 (TCF/LEF1), recruits co-activators such as CREB-binding protein (CBP) and E1A-binding protein p300 (p300), and activates the transcription of target genes, including ATP-binding cassette sub-family B member 1 (ABCB1), MYC proto-oncogene (MYC), neuroblastoma-derived v-myc avian myelocytomatosis viral related oncogene (MYCN), among others. Notably, mutations activating this pathway are more common in metastatic prostate cancer than primary tumors. Research shows WNT/β-catenin activation strongly connects to cancer cell growth, invasion, bone spread, drug resistance, and neuroendocrine transformation in advanced disease. Scientists have developed various approaches to target this pathway. One strategy uses antibodies and molecules targeting WNT proteins and receptors, such as Foxy-5, which mimics WNT-5A, and vantictumab, which targets multiple FZD receptors. Another approach involves WNT secretion inhibitors that target the porcupine enzyme, including compounds like IWP-2 and ETC-159. Researchers have also developed drugs like ICG-001 and PRI-724 that block the interaction between β-catenin and CBP. However, targeting the WNT pathway faces several significant challenges. The pathway's complexity, involving numerous proteins and receptors, makes it difficult to effectively block. The interactions between different WNT signaling routes are not fully understood, complicating treatment approaches. Additionally, since WNT signaling plays crucial roles in normal tissue maintenance, blocking it can cause widespread side effects. Future research aims to address these challenges through several approaches. Scientists are exploring combination therapies to enhance effectiveness while reducing side effects. They're working to better identify which patients might respond best to WNT-targeting treatments. Researchers are also deepening their understanding of how different WNT pathways interact and developing more precise targeting approaches that could minimize side effects. This comprehensive approach to understanding and targeting the WNT pathway represents a promising but challenging area in prostate cancer treatment [225], [226].

### Targeting other pathways

#### Vascular endothelial growth factor (VEGF)

VEGF drives new blood vessel formation, which is critical for tumor survival and growth by ensuring oxygen and nutrient supply. The VEGF family includes four proteins (A through D) that bind to three different receptors (VEGFR1-3), triggering pathways crucial for cancer cell survival and movement. VEGFA, particularly abundant in prostate cancer, correlates with disease progression and recurrence. While bevacizumab (anti-VEGFA therapy) combined with docetaxel showed improved progression-free survival and PSA response, it didn't significantly extend overall survival in metastatic castration-resistant prostate cancer (mCRPC) [227], [228].

#### Endothelin A receptor (ETAR)

ETAR signaling, activated by endothelin-1, influences cancer progression through multiple mechanisms, including blood vessel formation and bone tissue changes. Two ETAR inhibitors, zibotentan and atrasentan, showed initial promise in phase 2 trials but failed to demonstrate significant survival benefits in larger phase 3 studies. However, patients with elevated bone metabolism markers showed notable survival improvement with atrasentan treatment [155], [229], [230], [231].

#### Transforming growth factor beta (TGF-β)

TGFβ regulates cell development and movement through a complex signaling cascade involving SMAD proteins. Higher levels of TGFβ or its downstream targets indicate more aggressive disease and poorer outcomes. TGFβ particularly affects bone metastasis and immune system suppression. New treatments targeting this pathway include galunisertib (a TGFβR1 inhibitor) and M7824 (targeting both TGFβ and PD-L1), which are currently under clinical evaluation [232], [233], [234], [235].

#### RTKs and nonreceptor tyrosine kinases (NRTKs)

In prostate cancer development and progression, two key protein families − receptor tyrosine kinases (including FGFR, EGFR, PDGFR, and VEGFR) and nonreceptor tyrosine kinases (like SRC) − emerge as critical players and therapeutic targets. When activated by specific molecules or stimuli, these kinases undergo a process called cross-phosphorylation at tyrosine sites, triggering important cellular pathways like PI3K/AKT, phospholipase C, and Janus tyrosine kinase. These pathways then influence how genes controlling cell growth, survival, and development are expressed. The FGF system, particularly FGFR1/4 receptors and their corresponding FGF molecules (types 1,2,4,8, and 17), shows elevated presence in prostate cancer tissue. When overactive, this system promotes several cancer-supporting processes: tumor growth, blood vessel formation, cellular transformation, and increased androgen receptor expression. This pathway becomes especially relevant in androgen receptor-negative cancers and treatment-resistant cases. A new drug called erdafitinib, which blocks all FGFR types, is currently under investigation for treating prostate cancers lacking both androgen and neuroendocrine markers. Changes in EGFR, whether through mutation or increased expression, often signal more aggressive disease with higher likelihood of spread and return. While EGFR-blocking drugs like gefitinib showed limited success in clinical studies, another drug called cetuximab demonstrated promising results, particularly in patients whose tumors had high EGFR levels. SRC activity increases dramatically in prostate cancer that has spread to bones. Despite early promise, combining SRC inhibitors (dasatinib or saracatinib) with standard treatments hasn't significantly improved patient survival, though some delay in disease progression was observed. Additionally, drugs targeting multiple kinases simultaneously (such as dovitinib, sunitinib, and cabozantinib) have shown varying degrees of effectiveness in clinical trials, with some showing better results in specific patient groups but generally requiring further investigation to determine their optimal use [236], [237], [238], [239], [240], [241], [242], [243], [244], [245].

#### MEK

The MAPK signaling pathway becomes abnormally active due to mutations in upstream components like RAS and RAF, along with growth factor influences, all converging at MEK. In advanced prostate cancer, heightened activity of the RAS/RAF/MEK/ERK cascade indicates poorer outcomes and resistance to androgen-targeted therapies. Scientists have observed elevated levels of MAPK pathway proteins and activated ERK1/2 in mCRPC. This has led to clinical testing of trametinib, a MEK1/2 inhibitor, as a potential treatment for mCRPC patients [246], [247], [248].

#### Modulation of alternative splicing

Nearly all human genes undergo alternative splicing, a process that creates different protein variants with distinct roles. In prostate cancer, this mechanism affects several key genes, including FGFR, ERG, VEGFA, and AR. The FGFR2 gene illustrates this complexity − its splicing produces two variants: FGFR2(IIIb) in epithelial cells and FGFR2(IIIc) in mesenchymal cells. This switch serves as an indicator of cancer cells transitioning to a more aggressive state. Research has identified several compounds that can influence this splicing pattern, including calcium channel blockers and opioid antagonists. Another example involves the ERG gene, which fuses with TMPRSS2 in half of prostate cancer cases. Scientists have developed molecules that can alter ERG splicing, showing promising results in reducing tumor growth in laboratory studies [155].

#### c-MET

Regarding c-MET targeting, researchers recognize that prostate cancer treatment requires combined approaches with anti-androgen therapy. Clinical studies have explored various drug combinations. One trial examining crizotinib with enzalutamide revealed significant interactions between the drugs, while another study showed improved outcomes when combining cabozantinib with androgen deprivation therapy. Tivantinib demonstrated modest benefits with manageable side effects in certain mCRPC patients. Recent research suggests that simultaneously targeting c-MET and PARP might offer additional benefits, as this combination effectively suppressed cancer cell growth and spread in laboratory studies, though clinical testing is still needed [249], [250], [251].

### Testicular germ cell tumors

Germ cell tumors (GCTs) predominantly originate from early reproductive cells, with approximately 95 % developing from primitive germ cells or gonocytes, making them distinct among cancers. These tumors can be divided into two main categories: seminomas and nonseminomas. The latter category includes various tumor types showing different developmental patterns, such as embryonal carcinomas, choriocarcinomas, yolk sac tumors, and teratomas. GCTs stand out for their remarkable response to chemotherapy compared to other adult solid tumors, achieving overall cure rates exceeding 90 %, and successful treatment rates of 70–80 % even in cases where the cancer has spread. When standard platinum-based therapies and intensive chemotherapy fail, treatment becomes more challenging. Individual conventional drugs have demonstrated limited effectiveness, with response rates ranging from 10 % to 37 % and few complete recoveries. Yet, since these patients tend to be young, generally healthy, and have good organ function, doctors often combine multiple active medications. This approach has yielded better results, with response rates between 40 % and 60 %, and complete recovery in 5–30 % of cases [252], [253], [254], [255].

**Fig. 4:**
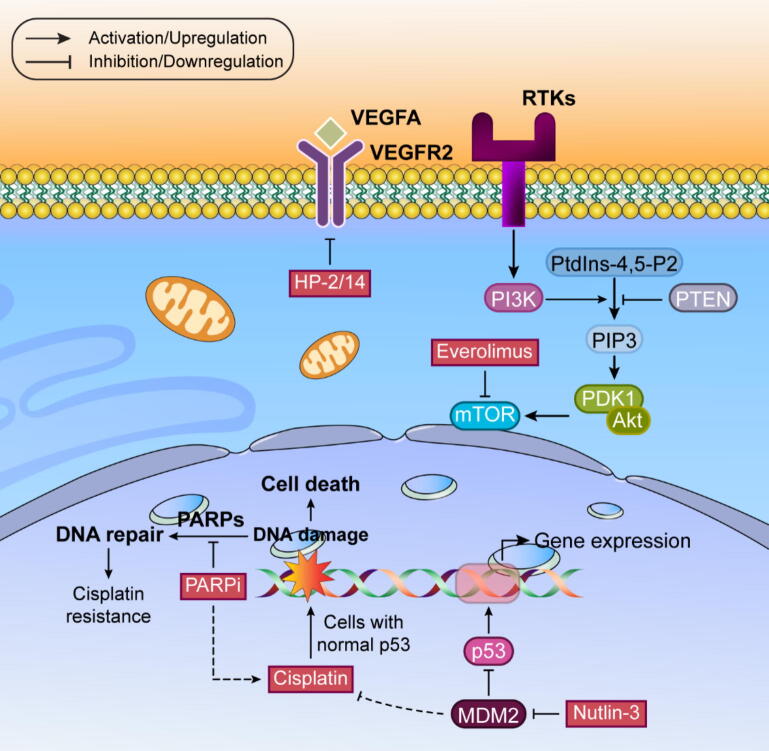
Targeted therapies and clinical trials for testicular germ cell tumors.

Research has uncovered various potential therapeutic targets in GCTs, including the p53-MDM2 interaction, various receptor tyrosine kinases (VEGFR, PDGFRβ, and KIT), MAPK signaling, DNA repair mechanisms, cell surface markers like CD30, and immune system regulators such as the PD-1/PD-L1 pathway (Fig. 4). While many targeted treatments have been tested in resistant GCT cases over recent years, their effectiveness has been modest, with few significant responses [256]. It's worth noting that most clinical trials did not select participants based on specific biological markers that might predict treatment success.

#### The p53–MDM2 interaction

The protein p53 acts as a crucial tumor suppressor by inhibiting growth and promoting cell death, but its function is often compromised in cancer cells. In many tumors, excessive production of MDM2, which specifically targets p53 for degradation and blocks its transcriptional activity, effectively neutralizes p53′s protective role. The high effectiveness of cisplatin treatment in germ cell tumors (GCTs) has been attributed to their retention of normal p53, which enables a strong cell death response when DNA is damaged by cisplatin. While p53 mutations are uncommon in untreated GCTs, suggesting an inadequate initial response to malignant transformation, about 25 % of cisplatin-resistant cases show either p53 mutations or increased MDM2 levels, resulting in reduced cell death capability. Studies have shown that persistent p53-MDM2 binding prevents cell death in cisplatin-resistant GCT cell lines. Targeting MDM2 and MDMX to activate p53 could offer an alternative treatment approach for chemotherapy-resistant GCTs. For instance, the MDM2-blocking compound nutlin-3 has successfully restored cisplatin sensitivity in resistant cells through non-DNA-damaging p53 activation, though clinical trials of such inhibitors haven't yet begun in GCT patients. The retinoblastoma pathway regulates cell division through various proteins including cyclin-dependent kinase inhibitors and activators, the Rb protein, and E2F transcription factors. DNA damage triggers the G1-S checkpoint to pause cell division and allow DNA repair. GCTs typically show an abnormal chromosome 12 configuration containing genes that control this checkpoint, including CDK4 and cyclin D2, which are often overexpressed. Most GCTs show reduced Rb protein levels, except in differentiated components like teratomas. While Rb mutations are rare in GCTs, mature teratomas express high Rb levels that promote growth when activated by CDK4/6 [257], [258], [259], [260].

Clinical trials with CDK4/6 inhibitors have shown promise, with ongoing studies of new compounds like ribociclib in resistant teratomas. The DNA damage response (DDR) in GCTs differs from other solid tumors, showing low baseline activation and few mutations in key DDR genes. Cisplatin treatment triggers DDR activation by causing DNA cross-links. While GCTs can repair some types of DNA damage, they struggle with certain cross-links that lead to double-strand breaks, which can be fatal to cells. This deficiency in homologous recombination repair makes GCTs potentially vulnerable to PARP inhibitors, similar to BRCA-mutated cancers. While PARP is overexpressed in many GCTs, particularly seminomas and embryonal carcinomas, its levels don't correlate with patient outcomes. Laboratory studies show PARP inhibitors can enhance cisplatin's effects and may work independently, particularly in resistant cases. Current clinical trials are investigating whether GCTs respond to PARP inhibitors like olaparib, regardless of PARP expression levels [261].

#### Growth factor receptors/receptor tyrosine kinases and ligands

Growth-promoting pathways such as PI3K-AKT and MAPK are triggered by receptor tyrosine kinases. Studies have shown that treatment-resistant germ cell tumors (GCTs) often express several of these receptors, including VEGFR2, PDGFRβ, c-Kit, and EGFR. GCTs produce significantly higher levels of VEGF and PDGF compared to normal testicular tissue. The elevated presence of VEGF and VEGFR2 suggests that drugs targeting blood vessel formation might be effective in resistant GCTs. In resistant GCT cell lines, increased PDGFRβ leads to enhanced AKT signaling. While drugs like sunitinib, sorafenib, and pazopanib that target multiple kinases showed promise in laboratory studies, their clinical effectiveness has been limited. Sunitinib achieved modest success with temporary partial responses in 13 % of patients, while pazopanib and sorafenib only showed minor effects on tumor markers and disease stabilization. New compounds targeting VEGFR2 (HP-2 and HP-14) have shown early promise in laboratory testing, particularly HP-14′s ability to restore cisplatin sensitivity. The ErbB family of receptors, including EGFR, Her2, ErbB3, and ErbB4, represents another potential therapeutic target. Certain components of nonseminomas show high EGFR expression, with one study finding EGFR overexpression in 88 % of resistant GCTs. However, targeting EGFR alone proved ineffective in animal studies, while dual inhibition of EGFR and Her2 showed more promise. Her2 overexpression occurs in 5–20 % of GCTs, with gene amplification in about 5 % of cases. The KIT receptor, crucial for early germ cell development, is frequently expressed in seminomas, with activating mutations present in 10–40 % of cases. These mutations, particularly in exon 17, often make tumors resistant to imatinib treatment. Clinical trials with imatinib have shown limited success, though individual cases have reported significant responses. The MET receptor, which activates important growth pathways, has also been investigated. Despite encouraging laboratory results, clinical trials with the MET inhibitor tivantinib showed poor results in resistant GCT patients, with minimal disease stabilization and brief progression-free survival. These findings highlight the complexity of targeting receptor tyrosine kinases in GCTs, where laboratory promise often hasn't translated into clinical success, suggesting the need for more refined approaches and possibly combination strategies [262], [263], [264], [265], [266], [267], [268], [269].

#### Vascular endothelial growth factor

Blood vessel formation in tumors (angiogenesis) is crucial for cancer progression and spread. VEGF, produced by cancer cells, plays a key role in this process. Studies have found that about 41 % of germ cell tumors (GCTs) produce VEGF, which correlates with blood vessel development and helps predict cancer spread. Clinical trials have explored bevacizumab, an antibody targeting VEGF, in different combinations. When used with oxaliplatin, about one-third of patients showed improvement, though these effects typically lasted only 5 months. A more intensive treatment combining bevacizumab with multiple chemotherapy drugs showed better response rates (89 %), but this study included patients with varying disease severity and had concerning safety issues, including four deaths. While one patient with growing teratoma syndrome showed improvement with bevacizumab alone, there isn't enough evidence to recommend its use in GCT treatment. The PI3K-AKT pathway appears important in GCT growth and treatment resistance. This pathway can be activated by various receptors, and resistant cancer cells often show increased AKT activity linked to higher PDGFRβ levels. About 13 % of resistant GCTs have PI3K and AKT1 mutations, while 50–60 % show reduced PTEN, which normally helps control this pathway. While drugs targeting this pathway exist, they haven't been thoroughly tested in resistant GCTs. Changes in the MAPK pathway, including mutations in Ras proteins, occur in 7–25 % of resistant GCTs, particularly in seminomas. The BRAF V600E mutation has been found in some resistant tumors, though its importance remains unclear. Current research is exploring combination treatments targeting BRAF and MEK in rare cancers with BRAF mutations. The mTOR protein, which controls cell growth and survival, has emerged as another potential target. While seminomas show high levels of mTOR-related proteins, clinical trials with the mTOR inhibitor everolimus have shown limited success, with no significant tumor shrinkage but some ability to temporarily stop disease progression. Despite identifying numerous potential molecular targets and promising laboratory results, targeted therapies haven't yet proven effective enough for clinical use in resistant GCTs. The potential of newer immunotherapy approaches, such as PD-1/PD-L1 inhibitors, is still being investigated [270], [271], [272], [273], [274].

### Renal carcinomas

Kidney cancer represents a significant health challenge in the urinary system, showing concerning trends in both occurrence and death rates worldwide. Statistics from 2020 indicate approximately 431,000 new diagnoses and 179,000 deaths globally. For 2023, projections from the National Cancer Institute suggest kidney and renal pelvis cancer will comprise 4.2 % of new cancer cases, with an estimated 81,000 new diagnoses. Renal cell carcinoma (RCC) makes up about 90 % of kidney cancer cases. As one of the top ten most common cancers globally, RCC stands out among urogenital cancers for its particularly high mortality rate. Its global recurrence and death rates exceed 40 %, with men showing higher incidence rates (sixth most common) compared to women (tenth most common). RCC manifests in several forms, with clear cell RCC (ccRCC) being most prevalent (70–80 %), followed by papillary RCC (10–15 %) and chromophobe RCC (5–10 %). Less frequently observed variants include collecting duct, sarcomatoid, and unclassified types. Each variant demonstrates unique genetic profiles and molecular characteristics, with ccRCC typically showing the most aggressive behavior, particularly when involving blood vessel invasion. Current treatment approaches combine cytokines, targeted therapies, and immune checkpoint inhibitors. However, single-drug treatments often lead to resistance, prompting research into combination therapies. Yet, even combined approaches have limitations, highlighting the need for new therapeutic strategies, particularly for patients who don't respond well to existing treatments or develop resistance [275], [276], [277], [278], [279], [280], [281].

**Fig. 5:**
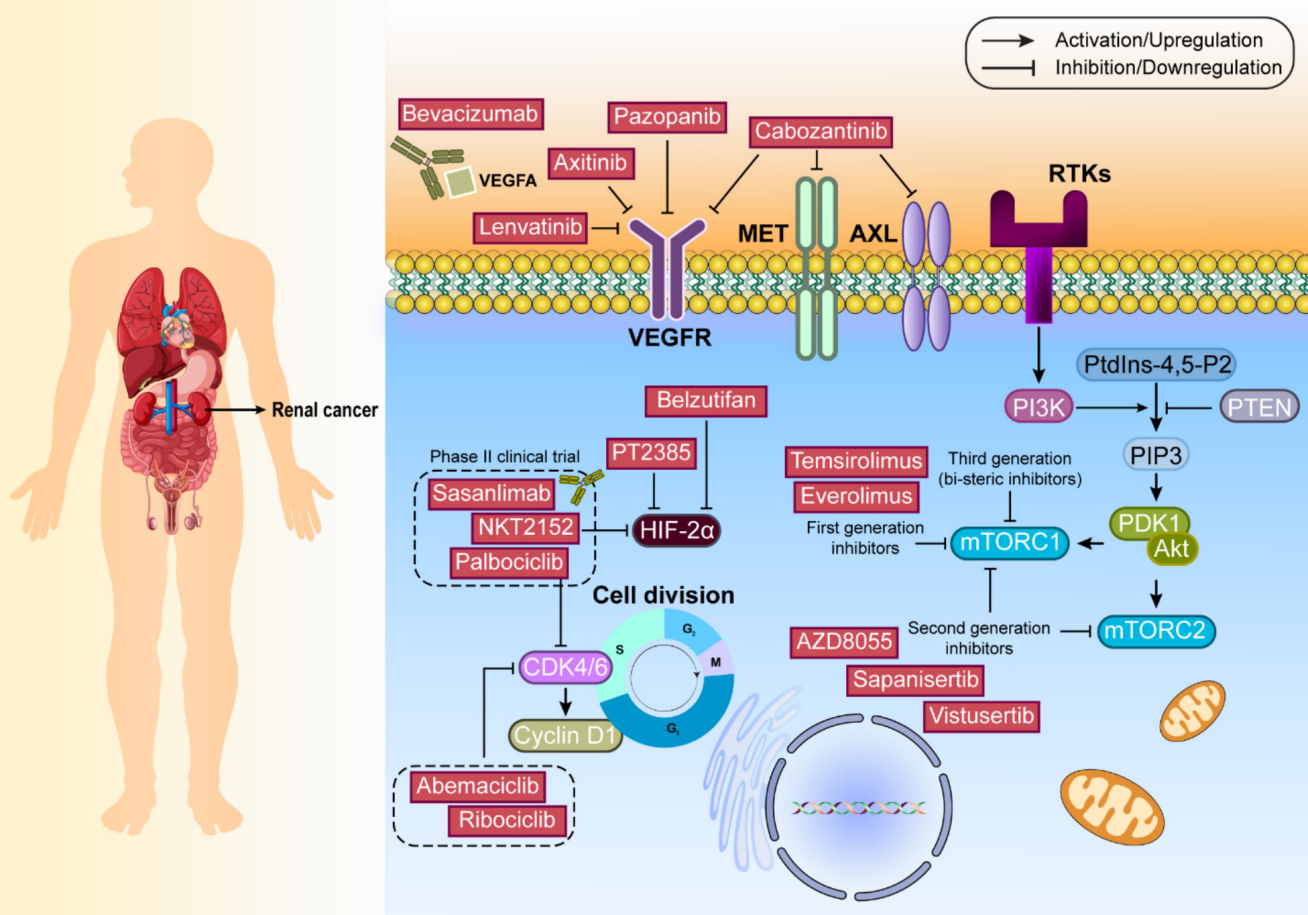
Targeted therapies and clinical trials for renal cancer.

The development of targeted therapies in RCC has been influenced by understanding the role of on Hippel–Lindau tumor suppressor (VHL) mutations, which increase HIF activity and subsequently VEGF expression, promoting blood vessel formation in tumors (Fig. 5). The mTOR pathway's involvement in HIF activation has also been recognized. Consequently, current targeted treatments primarily focus on the VEGF and mTOR pathways, though continued research seeks to identify new therapeutic targets and approaches. This ongoing research is crucial for expanding treatment options, addressing drug resistance, and advancing personalized medicine approaches in RCC treatment [282], [283], [284].

#### TKIs

TKIs play a fundamental role in treating RCC by targeting RTKs to suppress tumor blood vessel formation and growth. The approved treatments include first-line options like sunitinib, pazopanib, axitinib, lenvatinib, and cabozantinib, while sorafenib and tivozanib serve as second-line treatments for clear cell RCC, and erlotinib specifically targets papillary RCC [285], [286].

Clinical studies have demonstrated that sunitinib alone outperforms interferon-α in extending both progression-free and overall survival. Pazopanib has shown effectiveness in both newly diagnosed and previously treated metastatic RCC patients, offering better quality of life compared to sunitinib. Axitinib, which targets three VEGF receptors, has proven more effective than sorafenib in patients who previously received sunitinib. Recent developments have produced more sophisticated multi-target TKIs. Lenvatinib, for instance, targets multiple receptors involved in blood vessel formation and tumor growth. Its combination with everolimus has shown remarkable results, significantly extending progression-free survival. Cabozantinib, targeting MET, VEGF, and AXL, has demonstrated effectiveness in patients resistant to standard treatments, showing improved survival rates and treatment response compared to existing options. Bevacizumab, an antibody targeting VEGF, has shown promise when combined with interferon or atezolizumab, particularly in specific patient subgroups. Despite their effectiveness, TKIs come with significant challenges. Common side effects include skin problems, high blood pressure, digestive issues, and damage to mucous membranes. Severe adverse reactions are frequent, with one study reporting serious complications in 78 % of sunitinib-treated patients. Drug resistance remains a major concern with TKIs, attributed to various mechanisms including reduced drug availability due to cellular sequestration, increased tumor invasiveness through epithelial–mesenchymal transition (EMT), enhanced blood vessel protection, greater inflammatory cell recruitment, and genetic variations affecting drug response. Given these challenges, TKI monotherapy is typically reserved for specific cases, such as patients who cannot tolerate immunotherapy or prefer oral medication. While TKIs have advanced RCC treatment significantly, the field continues to seek solutions for drug resistance and side effect management [287], [288], [289], [290], [291], [292], [293], [294].

#### mTOR inhibitors

In healthy cells, the VHL protein helps regulate mTORC1 signaling by controlling the breakdown of proteins involved in the mTOR pathway. However, in RCC, defective VHL function leads to increased mTOR pathway activity. This process creates a feedback loop where elevated HIF levels activate the AKT-mTOR pathway, enhancing cancer cell survival, while activated mTOR further increases HIF expression, driving disease progression. This understanding has led to the development of mTOR inhibitors as a therapeutic strategy for RCC [293], [294].

mTOR inhibitors are classified into three generations. The first generation includes non-competitive inhibitors that specifically target mTORC1. Second-generation drugs are ATP-competitive inhibitors that affect both mTORC1 and mTORC2. The third generation consists of bi-steric inhibitors that selectively target mTORC1 through a different mechanism. Among first-generation inhibitors, everolimus and temsirolimus are most commonly used in RCC treatment. Everolimus has proven effective in patients who don't respond to or cannot tolerate anti-VEGFR treatments, doubling progression-free survival compared to placebo. Temsirolimus has shown superior results compared to interferon-α in advanced RCC, improving both overall survival and progression-free survival. It has been particularly effective in treating various non-clear cell RCC subtypes, demonstrating good tolerability and disease control rates, especially in Asian populations with intermediate or moderate risk. The development of second-generation inhibitors like AZD8055, sapanisertib, and vistusertib addresses the limitation of mTORC2′s role in drug resistance, which first-generation drugs cannot fully combat. However, these newer agents, while showing promising anti-tumor effects, have not yet received regulatory approval and carry significant toxicity concerns. Third-generation mTOR inhibitors represent the latest advancement, offering more targeted mTORC1 inhibition through innovative spatial configuration approaches. These newer drugs show promise for future clinical applications due to their more sustained therapeutic effects and potentially improved safety profiles [295], [296], [297], [298], [299], [300].

#### CDKs inhibitor

The development of CDK inhibitors emerged as a response to diseases caused by CDK dysfunction. Given that most cancers exhibit irregular cell growth and division patterns, CDK inhibition has become an appealing therapeutic strategy, leading to extensive research and development of various inhibiting compounds [301], [302]. In the context of RCC, only select CDK inhibitors have demonstrated meaningful therapeutic potential. Notable among these is ribociclib, which specifically targets CDK4/6. Research has shown that ribociclib can effectively suppress RCC growth and trigger cancer cell death both in laboratory settings and living organisms. Importantly, it shows enhanced effectiveness when combined with chemotherapy or immunotherapy treatments, while sparing healthy kidney cells and connective tissue cells from damage. Research has also revealed promising results when combining CDK4/6 inhibitors with drugs that target HIF-2α, particularly in RCC cases lacking the VHL gene. Current clinical investigations include a study examining how patients with advanced RCC respond to combined treatment with abemaciclib and sunitinib, focusing on safety aspects and optimal dosing. Additionally, an ongoing phase II clinical trial is investigating a three-drug combination approach: the HIF-2α inhibitor NKT2152, the CDK4/6 inhibitor palbociclib, and the immunotherapy drug sasanlimab. This study aims to determine the most effective and safe dosage for patients with advanced clear cell RCC who have undergone previous treatments [303], [304].

#### HIF inhibitors

The alpha subunit of hypoxia-inducible factor (HIF-α) plays a crucial role in cancer progression by controlling genes that regulate blood vessel formation and glucose metabolism, such as VEGF and glucose transporter protein-1. Among the three HIF variants, HIF-2α has been identified as the primary driver of RCC, making it an important therapeutic target. Initial clinical trials with PT2385, the first HIF-2α inhibitor, showed promising safety results in patients with advanced clear cell RCC who had previously undergone multiple treatments. The response rates were encouraging, with over half the patients achieving disease stability and some showing partial or complete responses. However, inconsistent drug absorption posed challenges, leading to suboptimal treatment in certain patients. Belzutifan, a more refined second-generation HIF-2α inhibitor, has demonstrated broader effectiveness. A phase II trial involving patients with VHL disease showed positive results for both RCC and related tumors. Current research is exploring the combination of belzutifan with cabozantinib in previously treated clear cell RCC patients, with promising preliminary results [305], [306], [307], [308], [309].

Research has revealed that HIF-2α creates an environment that suppresses immune response through various mechanisms, including promoting regulatory T cell growth, increasing stem cell factor production, which leads to immunosuppressive cytokine release, and enhancing PD-L1 expression on tumor cells, which inhibits T cell function [310], [311]. These findings support combining HIF-2α inhibitors with immune checkpoint blockers. Laboratory studies indicate this combination approach can enhance anti-tumor immunity by improving T-cell infiltration and modifying immune cell composition within tumors. Several major clinical trials are currently underway, including the LITESPARK-022 study examining belzutifan plus pembrolizumab as post-surgery treatment and a comparative study of different combination therapies involving pembrolizumab, belzutifan, lenvatinib, and quavonlimab for initial treatment of advanced clear cell RCC. Notably, belzutifan has received FDA approval for treating VHL-associated cancers, including RCC [311], [312], [313].

#### c-MET

Cabozantinib functions as a dual inhibitor, targeting both VEGFR and c-Met pathways. Its ability to block blood vessel formation has established its role as a treatment option for metastatic Renal Cell Carcinoma (mRCC), particularly as a second-line therapy or as an initial treatment when immune checkpoint inhibitors aren't suitable. Research evaluating cabozantinib's effectiveness and safety profile has yielded promising results. An Italian multicenter study demonstrated strong therapeutic potential with minimal treatment discontinuation due to side effects. The study reported that about one-third of patients showed partial tumor response, another third maintained stable disease, and the remaining third experienced disease progression. Patients typically went eight months before their disease progressed, indicating the drug's clinical value. Additional research exploring c-Met expression patterns in mRCC patients receiving sunitinib treatment revealed that lower c-Met levels correlated with better survival outcomes. High c-Met expression emerged as a significant predictor of poorer progression-free survival, supporting the rationale for targeting this pathway in mRCC treatment [314], [315], [316], [317], [318], [319], [320].

While recent advances in targeted and immunotherapy approaches have significantly improved outcomes for renal cancer patients, several challenges persist, including development of drug resistance, management of treatment-related toxicity, and need for improved therapeutic strategies. Future research priorities should focus on understanding resistance mechanisms, discovering novel therapeutic targets and drugs, optimizing treatment combinations and dosing schedules, investigating the tumor microenvironment's role (including immune cell interactions, inflammatory responses, and metabolic changes), and identifying reliable biomarkers to predict treatment response, patient tolerance, and optimal drug selection. This comprehensive approach would enable more personalized treatment strategies and better toxicity management.

### Penile squamous cell carcinoma

Penile cancer represents an uncommon but highly aggressive type of urological cancer. Its rarity is evident in developed nations, where it affects roughly one in 100,000 individuals, placing it in the category of orphan diseases. The vast majority of cases (over 95 %) are squamous cell carcinoma (SCC), with various subtypes that differ in their outlook. Several factors increase the risk of developing penile cancer, including poor hygiene practices, long-term inflammatory conditions, HPV and HIV infections, presence or absence of circumcision, phimosis, and tobacco use. A major challenge in penile cancer management is the frequent delay in diagnosis, often exceeding six months, which results in many patients presenting with advanced disease when treatment options are limited. The primary treatment approach involves surgical removal of affected tissue. For high-risk localized cases, platinum-based combination chemotherapy may be administered before surgery. The disease is characterized by frequent local recurrence and spread to distant sites, leading to significant health complications and death rates. While early-stage cases can often be treated with penis-preserving surgery, advanced cases require a more comprehensive approach combining surgery with systemic treatments. However, the scientific evidence supporting current treatment guidelines is relatively limited. Additionally, existing imaging techniques cannot reliably detect lymph node involvement in high-risk patients without palpable nodes, despite approximately 25 % of such cases having microscopic lymph node spread [318], [319], [320], [321], [322], [323], [324].

Regarding treatment outcomes, one small retrospective study suggested improved survival in advanced cases (pN2/pN3) using combination therapy with vincristine, bleomycin, and methotrexate. More recent treatment approaches using combinations of cisplatin, fluorouracil, and either docetaxel or paclitaxel have shown promising results. Current recommendations for patients with confirmed lymph node spread (pN2 and pN3) include combination chemotherapy following radical lymph node removal, when feasible and when treatment aims are curative. For patients with advanced local disease, particularly those with fixed or large (>4 cm) lymph nodes or disease extending to nearby structures, combination therapy should be considered, with the goal of enabling subsequent surgical intervention in suitable candidates [322], [323], [324], [325], [326], [327], [328].

Traditional treatments like chemotherapy and radiation therapy show varying degrees of effectiveness, but lack strong randomized trial evidence supporting survival benefits and often come with significant side effects. Current research in urologic cancer treatment is exploring immunotherapy and targeted therapies through multiple clinical trials [329], [327]. These include three trials specific to penile SCC and six basket trials for rare cancers, focusing on CTLA-4 and PD-1/PD-L1 pathways to enhance immune system recognition of cancer cells. Treatment response depends on several factors including the tumor's immunogenic properties, immune cell presence within tumors, checkpoint inhibitor expression, and tumor susceptibility to immune attack. While microsatellite instability typically predicts immunotherapy effectiveness, it's rare in penile SCC. Instead, tumor mutation burden (TMB) serves as an alternative marker. Studies show that about 8 % of metastatic penile SCC cases have significant mutation levels, suggesting potential benefit from checkpoint inhibitor therapy. The immune response pattern in penile SCC typically shows higher immune cell concentration in surrounding tissue than tumor itself, presence of CD8+ T-cells in stromal tissue correlating with reduced lymph node spread, and negative impact of regulatory T-cells and M2 macrophages on survival. Research indicates that 50–60 % of penile SCC cases show PD-L1 expression on immune cells, with higher rates in HPV-negative tumors. This expression pattern correlates with poorer survival outcomes but suggests potential benefit from PD-1/PD-L1 inhibitor treatments [328], [329], [330].

Challenges in studying rare cancers like penile SCC include limited patient numbers for traditional clinical trials, rapid disease progression limiting treatment options, need for international collaboration, and difficulty in gathering statistically significant data. New research approaches include development of organoid models for treatment testing, centralization of treatment centers, international collaborative networks, and standardized investigation and treatment protocols. These innovative approaches aim to overcome the limitations of traditional clinical trials and advance treatment options. Patient-derived organoids particularly show promise for personalized treatment testing in rare cancers like penile SCC. The establishment of specialized treatment centers not only improves patient care but also facilitates research participation and standardized protocols [325], [331]. The field requires further investigation of additional immune checkpoints and continued development of novel research methodologies to address the unique challenges of studying and treating this rare but aggressive cancer.

## Limitations against targeted therapy: mechanisms of resistance

**Fig. 6:**
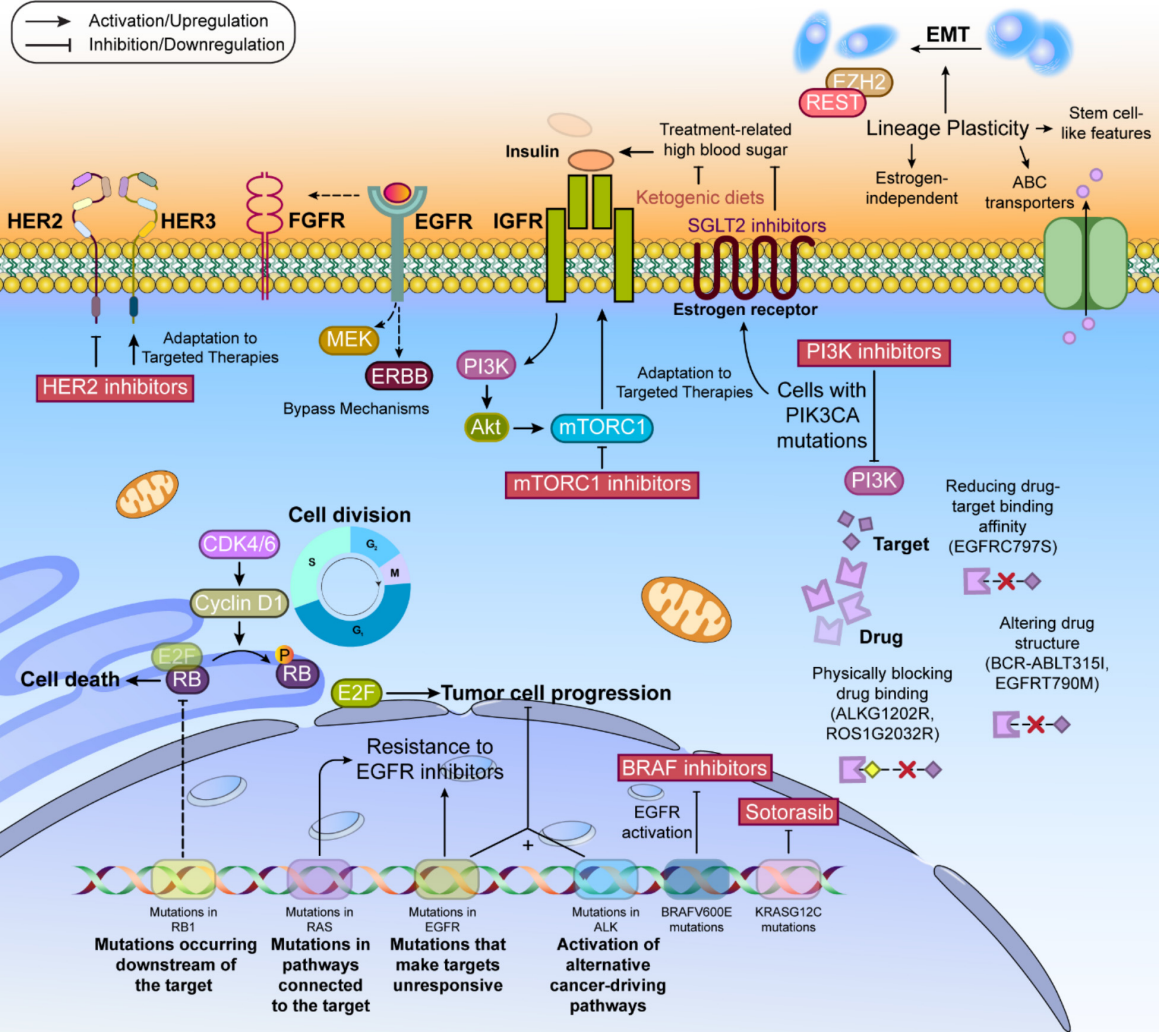
Mechanisms of resistance to targeted therapies.

Therapeutic failure of targeted treatments can occur for various reasons, including drug resistance, absence of the target, or insufficient drug exposure. This discussion focuses specifically on resistance mechanisms in cases where the target is both present and confirmed (Fig. 6).

The concept of primary resistance typically emerges from either a lack of target dependency or the presence of multiple targets within heterogeneous tumors. Several factors can indicate primary resistance including certain mutations that make targets unresponsive (Example: EGFR exon 20 insertions in lung cancer making tumors resistant to most EGFR inhibitors), alterations in pathways connected to the target (Example: RAS mutations affecting EGFR inhibition in colorectal cancer), mutations occurring downstream of the target (Example: RB1 mutations affecting CDK4/6 inhibitor effectiveness in breast cancer), and activation of alternative cancer-driving pathways (Example: simultaneous ALK and EGFR alterations in lung cancer). In rare cases of dual pathway activation, determining the dominant driver becomes crucial. For instance, in lung cancer, concurrent ALK and EGFR mutations appear in about 1.3 % of cases, typically within the same cell population. Evidence suggests EGFR mutations often dominate, as patients typically respond better to EGFR-targeted therapy. While protein phosphorylation levels might indicate treatment sensitivity, this approach still needs clinical validation [332], [333], [334], [335], [336], [337].

Gene silencing presents another challenge in targeted therapy. Even when genetic analysis identifies a targetable mutation, protein expression may be absent, rendering the treatment ineffective. Research examining 1,417 primary tumors revealed that approximately 13 % of genetic mutations fail to produce RNA transcripts. Some genes consistently show high transcription rates (like TP53, PIK3CA, and KRAS), while others frequently show lower rates (including ALK, CSF1R, and several others). Notably, tumors with higher mutation rates tend to show more silenced variants. Additional silencing can occur through various RNA modification mechanisms, including microRNA regulation and RNA interference [335], [336].

### Short-term adaptation to targeted therapies

When targeted therapies disrupt cellular feedback mechanisms, they can inadvertently trigger compensatory activation of upstream pathways. This phenomenon is observed in several contexts. In breast cancer with PIK3CA mutations, PI3K inhibition leads to increased estrogen receptor activity, reducing the effectiveness of single-agent treatments. Similarly, when mTOR and PI3K/mTOR inhibitors block mTORC1 and S6K, they eliminate important inhibitory signals that normally regulate insulin/IGFR and other receptor pathways. This disruption can lead to resistance through increased activation of PI3K, AKT, and ERK pathways, undermining the treatment's effectiveness. Treatment-related high blood sugar can trigger system-wide insulin increases, potentially activating PI3K signaling despite ongoing PI3K inhibitor treatment. Research has shown that managing blood sugar levels through ketogenic diets or SGLT2 inhibitors can help maintain treatment effectiveness by preventing this insulin feedback loop. Different cancers show varying responses to the same targeted treatments due to feedback mechanisms: BRAF inhibitors alone fail in colorectal cancer with BRAFV600E mutations due to EGFR activation, unlike in melanoma and lung cancer; KRASG12C-mutated colorectal cancer responds less favorably to sotorasib compared to lung cancer due to EGFR dependence; MEK inhibition can trigger PI3K/AKT activation through EGFR; and HER2 inhibitors can lead to HER3 upregulation. Protein translation control, particularly through the eIF4F complex, can influence resistance to various targeted therapies including HER2, BRAF, and MEK inhibitors. A notable example of rapid adaptation appears in KRASG12C inhibitor research, where cells can produce new, treatment-resistant KRAS proteins through EGFR-SHP2 and AURK-dependent processes [337], [338], [339], [340], [341], [342], [343], [344].

Acquired resistance typically develops through four main mechanisms: alterations affecting the drug target directly, alternative pathway activation, cancer cell transformation (including EMT and changes in cell type), and loss of target expression or dependency. These mechanisms highlight the complex nature of cancer treatment resistance and the need for comprehensive therapeutic approaches [342], [343], [344], [345], [346], [347].

### On-target mechanisms of resistance

Resistance to targeted therapies can develop through several mechanisms affecting the drug target directly. After treatment with tyrosine kinase inhibitors (TKIs), mutations often develop in the kinase domain, affecting drug effectiveness in three main ways: reducing drug-target binding affinity (example: EGFRC797S), altering kinase structure (gatekeeper mutations like BCR-ABLT315I, EGFRT790M), and physically blocking drug binding (solvent-front mutations such as ALKG1202R, ROS1G2032R). These mutations commonly affect membrane receptors and fusion proteins, but rarely intracellular kinases. Different drug structures may share resistance patterns. The specific fusion variant can influence resistance development, as noted with ALK variants. For antibody treatments, mutations can occur in protein extracellular domains, preventing antibody attachment. In hormone therapy, mutations affecting ligand binding regions can lead to constant receptor activation. Modified protein splicing can cause resistance through enhanced protein pairing (observed in 13–30 % of melanoma cases failing BRAF treatment) and continuous protein activation without ligand binding (as in prostate cancer). Resistance can develop through increased copies of mutated or normal genes (examples: EGFR, BRAF amplification), formation of extrachromosomal DNA carrying cancer genes, and dynamic regulation of gene copies based on treatment pressure. Resistance can emerge through changes in proteins with opposing functions, as demonstrated by PI3K and PTEN in PIP3 regulation. For instance, in breast cancers with PIK3CA mutations, PTEN loss can counteract PI3Kα inhibitor effects. Research suggests combining PI3Kα and PI3K p110β inhibitors might prevent this resistance. These various mechanisms underscore how cancer cells adapt to targeted treatments through multiple molecular strategies [345], [346], [347], [348].

### Bypass resistance mechanisms

Cell growth and proliferation are primarily controlled by three key signaling pathways: PI3K/AKT/mTOR, RAS/RAF/ERK (MAPK), and STAT/JAK. Different cancer-driving proteins interact with these pathways in various ways − some proteins like EGFR and HER2 activate two pathways (PI3K and MAPK), while others like ALK and ROS1 activate all three. BRAF uniquely signals through only the MAPK pathway. When cancer cells face targeted treatments, they can develop alternative signaling routes (bypass tracks) to maintain abnormal growth signals. These alternative paths become more common when stronger targeting drugs are used. Despite their diversity, these bypass mechanisms often follow similar patterns due to shared signaling networks. For receptor tyrosine kinase (RTK) inhibition, common resistance patterns include: In lung cancer: When EGFR is blocked, cells may activate MET, ERBB, or develop FGF2-FGFR1 mutations; When ALK is targeted, cells might increase EGFR activity or KIT levels; MET inhibition can lead to increased EGFR or HER2. Downstream pathway reactivation examples include: MAPK pathway activation through BRAF/NRAS/KRAS changes in lung cancer, MAP2K1/2 changes in melanoma during BRAF treatment, NF1 mutations in breast cancer resistant to hormone therapy, PI3K activation through various mechanisms in multiple cancer types. A notable recent discovery involves fusion genes appearing after treatment failure, particularly in lung cancer with EGFR mutations. These fusions occur more frequently with newer generation EGFR inhibitors (16 % vs 3 % with older drugs). RET fusions are most common (46 %), followed by ALK (26 %), NTRK1 (16 %), and FGFR3 (11 %). These fusion genes often involve unusual partner genes and have been observed in various cancers including colorectal, head and neck, and breast cancers, particularly after specific targeted treatments. This complex network of resistance mechanisms highlights how cancer cells can adapt to targeted therapies through multiple molecular strategies [348], [349], [350], [351], [352], [353], [354], [355], [356], [357], [358].

### Lineage plasticity

Cells can adapt to challenging conditions through phenotypic switching or cellular plasticity, allowing genetically identical cells to express different characteristics in response to environmental stresses like low oxygen, inflammation, or therapeutic interventions. This adaptation mechanism enables cancer cells to survive and grow without depending on their original cancer-driving factors, leading to treatment resistance. Evidence for non-genetic resistance comes from observations that resistant cells can become sensitive to drugs again after treatment breaks. This phenomenon involves several cellular changes. In Epithelial-Mesenchymal Transition (EMT), cells transform from epithelial to mesenchymal characteristics, driven by epigenetic changes, including increased Enhancer of Zeste Homolog 2 (EZH2) and RE1-silencing transcription factor (REST) activity, results in enhanced cell movement and treatment resistance, resistance mechanisms include reduced cell death proteins and increased drug removal through ABC transporters, and is observed in lung cancer cases resistant to EGFR inhibitors. In Cell Type Switching, breast cancer, AT-rich interaction domain 1A (ARID1A) mutations can cause cells to change from estrogen-dependent to independent types, and treatment-resistant cells often show stem cell-like features. In Cell Type Transformation, some lung cancers with EGFR mutations (3–14 %) transform from adenocarcinoma to squamous or neuroendocrine types after EGFR inhibitor treatment, similar changes occur in prostate cancer (about 17 %) after hormone therapy, and neuroendocrine transformation requires multiple changes including TP53 and RB1 gene inactivation, increased MYC and BCL2 protein levels, and enhanced AKT pathway activity. These adaptations represent complex cellular responses that allow cancer cells to evade targeted treatments through phenotypic rather than genetic changes [359], [360], [361], [362], [363].

### Loss of target or target dependencies

Cancer cells employ various resistance mechanisms to survive treatment through DNA repair mechanisms where tumors with DNA repair defects depend on PARP for survival and in BRCA1/2, PALB2, and RAD51C/D mutated cancers, cells can develop reversion mutations to restore protein function, affecting both PARP inhibitor and platinum treatment effectiveness, for example, ovarian cancer patients showing BRCA reversions in blood tests before rucaparib treatment typically have shorter progression-free survival. Target Loss occurs when PARP1 function can be lost through mutations or increased PARylation, and some breast cancers may lose estrogen receptors, making hormone therapy ineffective. Additional resistance strategies include Drug Efflux where increased P-glycoprotein pump activity can remove drugs from cells; Adaptive Mutation where some cancer cells temporarily increase mutation rates under stress, involving reducing DNA repair capabilities and increasing oxidative stress, reversing once resistance develops; Epigenetic Changes involving non-DNA sequence modifications that can cause resistance, creating “persister cells” that survive treatment through dormancy, showing high KDM5A expression affecting histone methylation; microenvironment factors where surrounding cells can release growth factors like HGF, providing resistance in various cancers through MET receptor activation, though some drugs like crizotinib can counter this by blocking multiple targets; and APOBEC-Driven Mutations where APOBEC enzymes modify DNA increasing genetic diversity, with APOBEC3B being active in over 50 % of cancers, creating ongoing mutations that drive cancer evolution and producing new cancer-driving mutations in cell subpopulations. These mechanisms show how cancers adapt through multiple sophisticated strategies to evade treatment effects [75], [348], [364], [365], [366], [367], [368].

## Combination therapies for enhanced efficacy

**Fig. 7:**
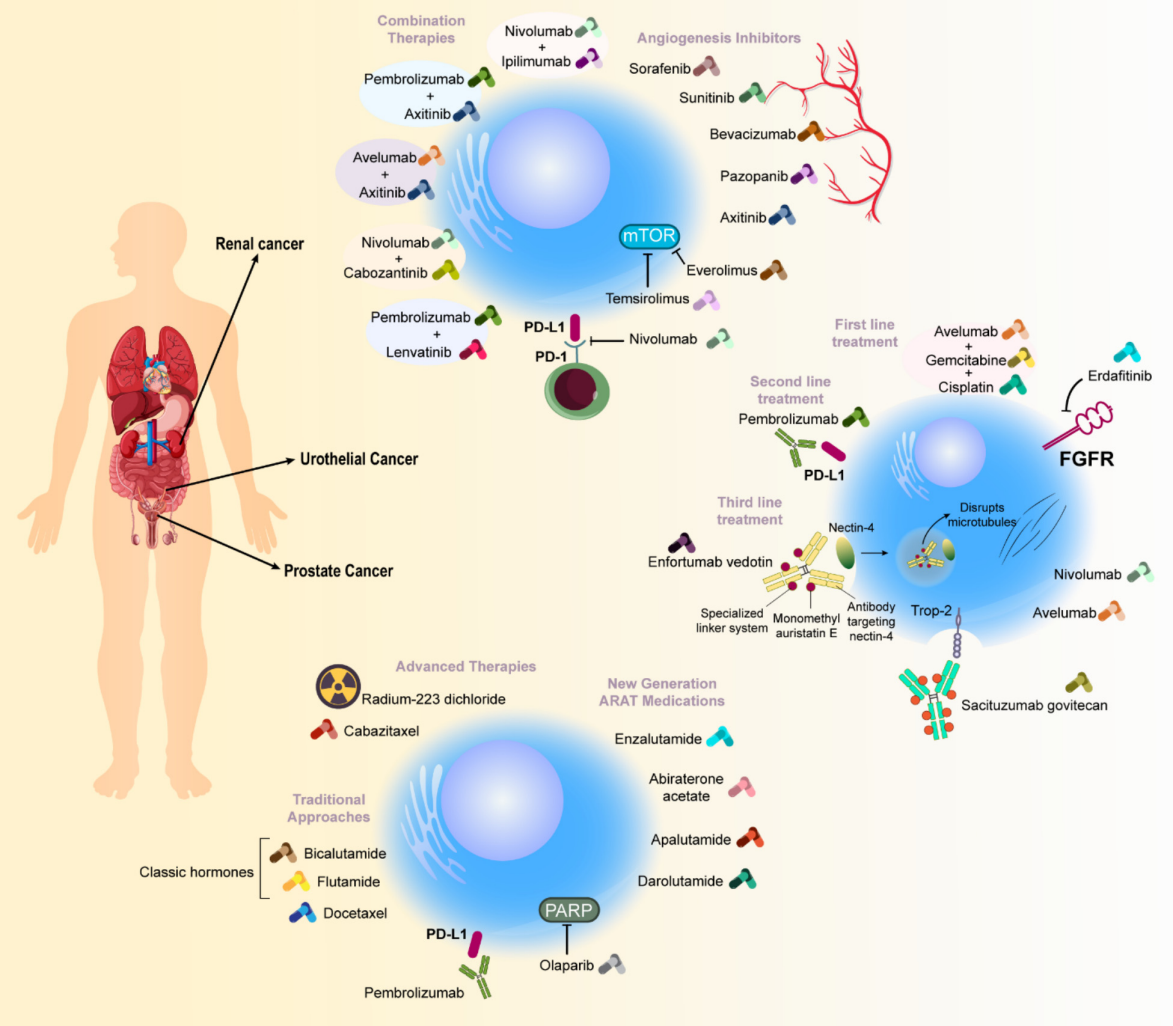
Combination therapies for urologic neoplasms.

The treatment landscape for metastatic urological cancers has traditionally involved distinct strategies for each cancer type. For example, RCC has typically been managed with TKIs, UC with cytotoxic chemotherapy, and prostate cancer with ADT. While these treatments remain important, new therapeutic options have emerged including ICIs that target multiple pathways including PD-1 (found on active T cells), PD-L1 and PD-L2 (present on cancer cells and antigen-presenting cells), and CTLA-4, which are now widely used across urological cancers. PARP Inhibition through Olaparib (Lynparza) received approval for treating castration-resistant prostate cancer with BRCA1/2 mutations. ADCs like Enfortumab vedotin (Padcev) represents a new approach that combines targeted antibody delivery with anti-cancer agents, uses a specialized linker system for drug release, and was recently introduced in Japanese medical practice. These developments mark a significant shift in treating metastatic urological cancers, with ongoing clinical trials exploring additional treatment possibilities. The field continues to evolve rapidly, transforming treatment approaches for these diseases (Fig. 7) [102], [112], [114], [121], [122].

### Renal cell cancer

The medical treatment of metastatic RCC has seen two major breakthroughs, with the First Breakthrough bringing Multiple Drug Approvals including Angiogenesis Inhibitors (Sorafenib/Nexavar, Sunitinib/Sutent, Bevacizumab/Avastin − unavailable in Japan, Pazopanib/Votrient, and Axitinib/Inlyta) and mTOR Inhibitors (Temsirolimus/Torisel and Everolimus/Affinitor). The Second Breakthrough introduced Immunotherapy with Single-Agent Treatment like Nivolumab (Optivo) that blocks PD-1 interactions and demonstrated effectiveness in Checkmate025 trial, and Combination Therapies including dual immunotherapy (Nivolumab + Ipilimumab) and mixed approaches (Pembrolizumab + Axitinib, Avelumab + Axitinib, Nivolumab + Cabozantinib, and Pembrolizumab + Lenvatinib) [287], [369], [370], [371], [372].

New Developments include Cabozantinib (Cabometyx) which targets VEGFR, MET, and AXL, proven superior to everolimus in second-line treatment (METEOR trial) and more effective than sunitinib in first-line setting (CABOSUN trial), while Pembrolizumab is showing promise as adjuvant therapy for high-risk non-metastatic RCC after surgery. Current treatment approaches typically involve combination therapies as first-line treatment, representing a significant evolution in RCC management [373], [374], [375], [376].

Current first-line treatment options for metastatic RCC include dual immunotherapy approach using Nivolumab + Ipilimumab which shows highest complete response rate (9 %), is particularly effective in sarcomatoid RCC cases, limited to IMDC intermediate/poor risk patients, and has higher disease progression rate (28 %), while Immunotherapy + TKI Combinations include Pembrolizumab + Axitinib with complete response rate of 5.8 % and lower disease progression (16 %), and Avelumab + Axitinib showing complete response rate of 4.4 % with disease progression around 15 %. Treatment Selection Considerations note that no definitive guidelines exist for choosing between options, ICI + TKI combinations are preferred for rapid disease control, and choice depends on individual patient factors. New Combination Approaches include Nivolumab + Cabozantinib (CheckMate 9ER trial) showing superior progression-free survival (16.6 vs 8.3 months), higher 12-month survival rate (85.7 % vs 75.6 %), better response rates (55.7 % vs 27.1 %), and manageable side effects, while Pembrolizumab + Lenvatinib (CLEAR trial) demonstrated significantly improved progression-free survival (23.9 vs 9.2 months), better overall survival rates, and notable side effects including hypertension and diarrhea. These developments establish combination therapy as the standard first-line treatment for metastatic RCC, with treatment selection individualized based on patient characteristics and disease presentation [377], [378], [379], [380].

### UC

Treatment Evolution in metastatic UC includes First-Line Treatment with traditional approach using Gemcitabine plus cisplatin (GC) combination chemotherapy and recent addition of Avelumab maintenance therapy following GC, based on successful Javelin Bladder 100 trial results targeting PD-L1. Second-Line Treatment involves Pembrolizumab immunotherapy, a humanized IgG4κ antibody targeting PD-1, approved for use after platinum-based chemotherapy failure and known for effectiveness and manageable side effects. Third-Line Innovation features Enfortumab vedotin, a novel Antibody-Drug Conjugate (ADC) with components including antibody targeting nectin-4 (common in epithelial cancers), drug using Monomethyl auristatin E (disrupts cancer cell microtubules), and specialized linker system ensuring targeted drug delivery, with a mechanism that induces cell-cycle arrest and cancer cell death. Future Developments show adjuvant nivolumab showing promise for non-metastatic high-risk muscle-invasive UC after surgery. This treatment sequence represents a significant advancement from traditional chemotherapy-only approaches, incorporating targeted therapies and immunotherapies for improved patient outcomes [114], [122], [381], [382], [383].

Clinical trial results and treatment advances show avelumab maintenance therapy in Javelin Bladder 100 study demonstrated overall survival of 21.4 months (with avelumab + BSC) vs 14.3 months (BSC alone), progression-free survival of 3.7 months vs 2.0 months, Grade 3 + adverse events of 47.4 % vs 25.2 %, and is recommended for patients responding to first-line GC therapy [384]. Enfortumab Vedotin Results in EV-301 Trial compared to standard chemotherapy showed improved survival of 12.88 vs 8.97 months, better progression-free survival of 5.55 vs 3.71 months, key side effects including maculopapular rash, fatigue, reduced neutrophils, and is established as effective third-line treatment [122]. New FDA-Approved Treatments include Erdafitinib, a FGFR1-4 inhibitor showing 40 % response rate in FGFR-mutated cases, 59 % response in post-immunotherapy patients, and manageable side effects with dose adjustments [385], while Sacituzumab Govitecan, a Trop-2 targeting antibody-drug conjugate, showed in TROPHY-U-01 results 27 % response rate, 5.4 months progression-free survival, with main side effects being blood count abnormalities [386]. Future Directions show Enfortumab vedotin plus pembrolizumab showing promise as first-line therapy with ongoing EV-302 trial comparing this combination to standard chemotherapy [380].

Adjuvant Nivolumab in CheckMate 274 for high-risk muscle-invasive disease showed improved disease-free survival of 21.0 vs 10.9 months, better results in PD-L1 positive patients, and maintained quality of life during treatment. Current Treatment Sequence (2022) includes First-line GC chemotherapy, Second-line Pembrolizumab, and Third-line Enfortumab vedotin, with treatment standards continuing to evolve through ongoing clinical trials evaluating new approaches [387].

### Prostate cancer

Evolution of prostate cancer treatment shows traditional approach using primary treatment with hormone therapy including androgen deprivation combined with ARAT agents, with early options limited to classic hormones (bicalutamide, flutamide) and Docetaxel for castration-resistant cases. Current Treatment Landscape in Japan includes New Generation ARAT Medications such as Enzalutamide (Xtandi), Abiraterone acetate (Zytiga), Apalutamide (Earleada), and Darolutamide (Nubeqa), along with Additional Advanced Therapies including Radium-223 dichloride (alpha radiation) and Cabazitaxel (enhanced taxane). Expanded Applications show several ARAT drugs now approved for hormone-sensitive metastatic cases with treatment adoption increasing due to effectiveness and tolerability [380].

Targeted therapy developments feature pembrolizumab approved for MSI-high tumors (2018) and olaparib as PARP inhibitor for BRCA1/2 mutated cases (2021). current challenge shows limited data on effectiveness and safety in Japanese patients with rare variants, despite universal healthcare coverage. This treatment landscape represents significant advancement from traditional hormone-only approaches, offering multiple options for various disease stages and genetic profiles [388], [389], [390].

Pembrolizumab Development and Approval received FDA authorization based on five pivotal trials (Keynote series) showing 39.6 % overall response rate, complete response in 7 % of cases, response duration of 1.6–22.7 months, with 78 % maintaining response beyond 6 months and consistent safety profile with previous studies, notable for being first biomarker-based approval regardless of cancer origin, though rare in prostate cancer cases [388], [389].

Olaparib Clinical Evidence from PROfound Trial for metastatic CRPC patients after ARAT therapy failure demonstrated progression-free survival of 7.4 vs 3.6 months, response rate of 33 % vs 2 %, overall survival of 18.5 vs 15.1 months, with main side effects being anemia and nausea, and showing particular effectiveness in BRCA1/2 mutations [102]. 2022 Treatment Standards include first-line options of ADT plus choice of Abiraterone acetate (high-risk only), Enzalutamide, or Apalutamide, and Docetaxel (newly approved based on CHAARTED trial). Sequential Approach involves after first-line progression BRCA1/2 testing where if positive, Olaparib is used, and if negative, docetaxel is considered, followed by later-line options including ARAT agents, Cabazitaxel, Radium-223, and Pembrolizumab for MSI-high cases [170], [391].

## Biomarker-specific targeted therapy in urologic malignancies

**Fig. 8:**
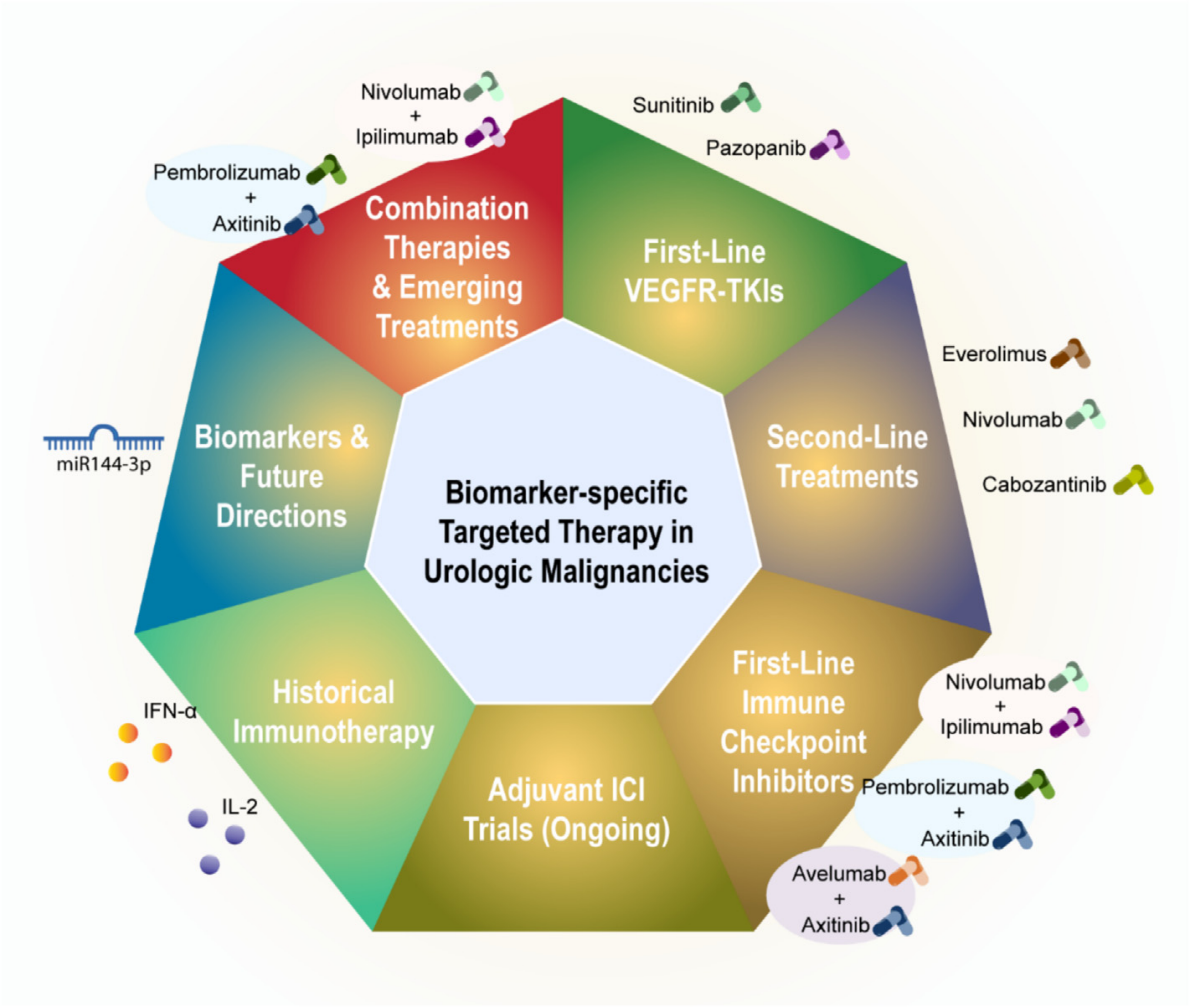
Biomarker-oriented targeted therapies against urologic malignancies.

Biomarker-based targeted therapies have recently attracted attention for urologic neoplasms (Fig. 8). Early Treatment Development reveals initial trials of VEGFR-TKIs (including sunitinib, sorafenib, pazopanib, and axitinib) in localized/advanced RCC showed limited survival improvements, leading to exploring immunotherapy options with four major Phase III studies now examining various immune checkpoint inhibitors post-surgery [392], [393], [394].

Historical Immunotherapy Context from 1990 s treatments relied on Interferon alpha showing modest 2.5-month survival benefit and Interleukin-2 demonstrating higher response rates (10–23 %) but significant toxicity. Molecular Understanding shows VHL gene inactivation, found in 75 % of clear cell RCC, drives VEGF overexpression and tumor blood vessel growth, leading to developing targeted TKIs. Key Clinical Studies include Sunitinib Trial with 750 untreated metastatic patients compared to interferon alpha showing improved progression-free survival of 11 vs 5 months, Pazopanib Study with 435 treatment-naive patients versus placebo extending progression-free survival to 11 vs 3 months, and COMPARZ Comparison involving 1110 patients comparing Sunitinib vs Pazopanib showing similar survival outcomes (29 vs 28 months) with Pazopanib showing better tolerability [287], [395], [396], [397].

Treatment Selection Approach demonstrates patient selection relied on clinical features rather than biomarkers, with benefits observed across different prognostic groups regardless of VEGFR expression levels. Evolution of Secondary Treatments shows mTOR Inhibition targeting mTOR-PI3K-AKT pathway (active in 66 % of cases) with RECORD-1 Study Results involving 416 patients post-VEGFR-TKI treatment comparing Everolimus vs placebo showing progression-free survival of 4.9 vs 1.9 months. Advanced Second-Line Options include Cabozantinib Development with multi-target approach (VEGFR, MET, AXL) where METEOR trial findings proved superior to everolimus with extended progression-free survival of 7.4 vs 3.8 months, and Immunotherapy Advancement using Nivolumab mechanism for PD-1 pathway blockade where CheckMate-025 trial with 821 previously treated patients demonstrated overall survival of 25 vs 20 months, though PD-L1 expression (24 % of cases) was not predictive. Treatment Guidelines Update (2016) for post-first-line VEGFR-TKI therapy established preferred options of Nivolumab and Cabozantinib, which replaced previous standards of Everolimus and secondary VEGFR-TKIs, representing significant improvement in managing treatment-resistant disease [398], [399], [400], [401].

Evolution in Metastatic RCC demonstrated through major clinical trials including CheckMate-214 Study with 1096 untreated patients comparing Nivolumab/Ipilimumab vs Sunitinib showing improved survival in intermediate/poor-risk groups and enhanced benefit with PD-L1 expression (28 % of cases), Keynote-426 Trial involving 861 patients testing Pembrolizumab/Axitinib combination with superior survival across all risk groups and benefits independent of PD-L1 status, and Javelin Renal 101 examining 886 participants (63 % PD-L1 positive) for Avelumab/Axitinib evaluation showing better progression-free survival and consistent benefits in PD-L1 positive cases (Table 3). Regulatory Impact led to FDA/EMA approval of all three combinations with treatment guidelines updated for universal approval of Pembrolizumab/Axitinib and Avelumab/Axitinib while restricting use of Nivolumab/Ipilimumab for intermediate/poor-risk cases [373], [377], [378], [379]. Biomarker Development shows MicroRNAs emerging as potential indicators with notable example of miR-144-3p's role in Sunitinib resistance, while research continues for comprehensive panels [402], [403].

**Table 3:** Clinical trials and treatment strategies for mRCC.

Category	Phase	**Population**	Intervention/comparison	Primary outcome	Results
First-line VEGFR-TKIs	III	750 patients with metastatic clear cell RCC (ccRCC)	Sunitinib (oral) vs. Interferon-alpha (IFN-α, subcutaneous 3x/week)	Progression-Free Survival (PFS)	Sunitinib showed significant PFS improvement over IFN-α [370],[406]
	III	435 treatment-naive patients with advanced/metastatic ccRCC	Pazopanib vs. Placebo (2:1 randomization)	Progression-Free Survival (PFS)	Pazopanib significantly improved PFS compared to placebo [395]
	III	1,110 patients with advanced/metastatic ccRCC	Sunitinib vs. Pazopanib	Progression-Free Survival (PFS) < br > Overall Survival (OS)	Comparable efficacy between sunitinib and pazopanib; pazopanib had a better toxicity profile [287]
Second-line treatments	III	416 patients with metastatic ccRCC previously treated with VEGFR-TKIs	Everolimus (10 mg/day, oral) vs. Placebo	Progression-Free Survival (PFS)	Everolimus significantly improved PFS as a second-line treatment [398]
	III	Metastatic ccRCC patients after VEGFR-TKI treatment	Cabozantinib vs. Everolimus	Progression-Free Survival (PFS)	Cabozantinib showed significant PFS improvement over everolimus [373]
	III	821 patients with advanced/metastatic ccRCC previously treated with 1–2 VEGFR-TKIs	Nivolumab (3 mg/kg IV every 2 weeks) vs. Everolimus (10 mg/day, oral)	Overall Survival (OS)	Nivolumab provided a significant OS benefit over everolimus [372]. FDA and EMA approved as second-line treatments
First-line immune checkpoint inhibitors	III	1,096 treatment-naive patients with metastatic RCC	Nivolumab + Ipilimumab vs. Sunitinib	Overall Survival (OS) in Intermediate/Poor-Risk Patients	Significant OS improvement with combination therapy in intermediate and poor-risk patients [377]
	III	861 treatment-naive patients with metastatic RCC	Pembrolizumab + Axitinib vs. Sunitinib	Overall Survival (OS)	Superior OS with pembrolizumab + axitinib across all IMDC risk groups, regardless of PD-L1 expression [379]. FDA and EMA approved for first-line treatment.
	III	886 treatment-naive patients with metastatic RCC	Avelumab + Axitinib vs. Sunitinib	Progression-Free Survival (PFS)	Significant PFS improvement with avelumab + axitinib, especially in PD-L1 positive patients [378]. FDA and EMA approved for first-line treatment.
Adjuvant ICI trials (Ongoing)	III	High-risk/locally advanced ccRCC (T2–4, N+)	ICIs as adjuvant treatment after surgery	Evaluate survival benefits as adjuvant therapy	Investigating the role of ICIs in the adjuvant setting following promising metastatic results [394],[407], [408], [409]
Historical immunotherapy	−	Metastatic RCC patients (1990s era)	Interferon-alpha (IFN-α) vs. Interleukin-2 (IL-2)	Response Rates and Overall Survival	IL-2 more potent but substantially more toxic compared to IFN-α [396],[410],[411]
Biomarkers & future directions	−	RCC patients	MicroRNAs (e.g., miR-144-3p) as biomarkers	Improve patient selection and treatment response	Potential for miRNA panels to guide RCC treatment decisions [402],[403]
Combination therapies & emerging treatments	Various	Various (Urothelial, Bladder, and Prostate Cancers)	Multiple ongoing trials (e.g., nivolumab + ipilimumab, pembrolizumab + axitinib, etc.)	Establish new first-line regimens and adjuvant therapies	Includes trials like CheckMate274, AMBASSADOR, Keynote-905/EV-303, IMvigor010 for urothelial cancer, ARASENS, KeyLynk-010 for prostate cancer. Liquid biopsies (e.g., FoundationOne Liquid) are emerging tools [412],[381],[413], [414], [415], [416], [417], [418], [419], [420], [421]

RCC with approved combination therapies including Nivolumab/ipilimumab, Pembrolizumab/axitinib, Avelumab/axitinib, Pembrolizumab/lenvatinib, and Nivolumab/cabozantinib, while research emphasis shifts toward post-nephrectomy treatment. Urothelial Cancer shows active clinical trials surrounding cystectomy with major ongoing Phase III studies including AMBASSADOR, Keynote-905/EV-303, and IMvigor010, while historical context shows MVAC regimen remains benchmark since 1985 alongside new immunotherapy trials comparing with GC protocol. Prostate Cancer Developments feature ARASENS trial evaluating darolutamide addition and KeyLynk-010 investigating olaparib/pembrolizumab combination, while 177Lu-PSMA-617 remains promising but pending clinical implementation. Emerging Diagnostic Approaches show shift toward liquid biopsies with advantages over traditional tissue sampling including higher success rate (tissue analysis failure: 30 %) and strong correlation with tumor tissue (81–92 % agreement), with FoundationOne Liquid now available in Japan, potentially revolutionizing genomic-based treatment selection [115], [116], [118], [124].

Urologic cancers, including clear cell RCC, bladder cancer, and prostate cancer, are systemic diseases that demand advanced diagnostic approaches that go beyond conventional methods. Multi-omics profiling and machine learning improve prognostic accuracy, treatment personalization, and risk assessment. Moreover, liquid biopsy, such as cell-free nucleic acid patterns, may provide a powerful non-invasive real-time monitoring of the disease. These innovations enhance precision medicine within the predictive, preventive, and personalized medicine (PPPM) framework, refining therapy selection and improving patient outcomes [404], [405].

## Future directions and concluding remarks

Modern advancements in urological cancer therapy highlight significant strides in targeted treatments, spurred by deeper molecular understanding and better insights into cancer biology. Central themes include the innovation of biomarkers that aim to surpass traditional indicators like PSA. This involves developing more precise prediction tools through genomic markers, protein analysis, and metabolic signatures. Additionally, integrated treatment strategies combine traditional chemotherapy, radiation, and immunotherapy, enhancing overall effectiveness, reducing drug resistance, and decreasing adverse effects, thus showing great potential to improve patient outcomes and care through tailored therapeutic combinations. Emerging therapeutic directions are exploring epigenetic research, focusing on gene regulation, studying drug resistance mechanisms, and testing new treatments, including HDAC inhibitor trials and novel epigenetic modulators. Advances in immunotherapy are also prominent, targeting current shortcomings in urological applications and aiming to refine therapies through new agents, combined treatments, and specific CAR-T therapies targeting PSMA/PSCA.

Diagnostic technology advancements have enhanced monitoring capabilities, enabling real-time response tracking and advanced imaging techniques such as PET scans and MRIs. A patient-centered approach is becoming increasingly important, integrating patient feedback, quality-of-life considerations, and comprehensive evaluations of outcomes, including survival rates, treatment experiences, and long-term health, all focused on developing more effective and patient-friendly protocols. At the molecular level, treatment advancements concentrate on specific targets, understanding intricate cancer pathways, and crafting personalized strategies. Key pathways under exploration include the PI3K/Akt/mTOR systems, MAPK signaling, growth factor mechanisms, c-Met/HGF pathway, PD-1/PD-L1 axis, and androgen receptors. Progress in immunotherapy highlights enhanced immune responses, successful applications in bladder cancer, synergies with traditional treatments, and better management of resistance. Overall, the focus remains on overcoming clinical translation challenges, continuing molecular research, studying resistance mechanisms, and pushing towards personalized treatments, indicating a promising future for individualized cancer care despite existing implementation and optimization challenges.

## Consent for publication

Not applicable.

## Compliance with ethics requirements

Not applicable.

## CRediT authorship contribution statement

**Kiavash Hushmandi:** Writing – review & editing, Writing – original draft, Resources, Investigation, Conceptualization. **Najma Farahani:** Writing – original draft, Investigation, Visualization. **Behzad Einollahi:** Writing – original draft. **Shokooh Salimimoghadam:** Visualization, Writing – review & editing, Resources. **Mina Alimohammadi:** Writing – original draft. **Liping Liang:** Writing – review & editing, Writing – original draft, Resources, Investigation, Conceptualization. **Le Liu:** Writing – review & editing, Writing – original draft, Resources, Investigation, Conceptualization, Supervision. **Gautam Sethi:** Supervision, Writing – review & editing.

## Ethics approval and consent to participate

Not applicable.

## Funding

This study was supported by NUHS Seed Grant from NUS Yong Loo Lin School of Medicine [NUHSRO/2022/090/T1/Seed-Sep/03] to GS.This work was also supported by the Shenzhen Science and Technology Program (Grant No. RCBS20221008093243060) and the Shenzhen Medical Research Fund (Grant No. A2403044).

## Declaration of competing interest

The authors declare that they have no known competing financial interests or personal relationships that could have appeared to influence the work reported in this paper.
